# Electronic Waste-Derived Nanomaterials: Environmental Release, Toxicity, and Public Health Risks

**DOI:** 10.3390/nano16140892

**Published:** 2026-07-20

**Authors:** Nargish Parvin, Keunhwan Park, Jae Hak Jung, Tapas K. Mandal

**Affiliations:** 1 Department of Mechanical Engineering, Gachon University, Seongnam 13120, Republic of Korea; nargish.parvin@gmail.com; 2 PiQuant Co., Ltd., Seoul 04323, Republic of Korea; 3 School of Chemical Engineering, Yeungnam University, Gyeongsan 38541, Republic of Korea; 4 Directorate of Research, Nehru Kendra, Mahatama Jyotiba Phule Rohilkhand University, Bareilly 243006, India

**Keywords:** electronic waste, nanomaterials, nanotoxicology, environmental pollution, public health, recycling, oxidative stress, circular economy

## Abstract

The rapid growth of electronic waste (e-waste) has emerged as a significant global environmental challenge, driven by increased consumption of electronic devices and shortened product lifecycles. E-waste contains a complex mixture of metals, polymers, and hazardous substances that, under physical, chemical, and biological processes, can transform into nanoscale materials. These electronic waste-derived nanomaterials are increasingly recognized for their potential environmental mobility, bioavailability, and toxicity. This review critically examines their generation pathways during recycling and environmental weathering, their release into environmental systems, and associated risks to ecosystems and human health. It also highlights current analytical approaches, regulatory challenges, and sustainable mitigation strategies.

## 1. Introduction

### 1.1. Global Expansion of Electronic Waste

Electronic waste (e-waste) has become one of the fastest-growing solid waste streams worldwide due to rapid technological advancement, digitalization, urban expansion, and increasing dependence on electronic devices in daily life [1,2]. The widespread use of smartphones, computers, wearable electronics, household appliances, batteries, photovoltaic systems, and internet-connected devices has significantly increased global electronic consumption during the past two decades. Current estimates indicate that annual global e-waste generation has exceeded 60 million metric tons, and future projections suggest continuous growth driven by accelerated technological turnover and consumer demand [3]. Despite increasing awareness regarding sustainable waste management, formal recycling infrastructure remains inadequate in many regions, resulting in substantial quantities of improperly handled electronic residues entering terrestrial and aquatic ecosystems. E-waste consists of highly heterogeneous materials including metals, plastics, semiconductors, glass, batteries, ceramics, and flame-retardant polymers [4]. Electronic devices contain valuable recoverable resources such as gold, silver, copper, palladium, lithium, cobalt, and rare earth elements that support large-scale recycling activities [5]. However, these materials also contain hazardous substances including lead, cadmium, mercury, chromium, arsenic, brominated flame retardants, and chlorinated compounds capable of causing severe environmental and biological damage during uncontrolled disposal or recycling [6]. Consequently, electronic waste represents both a valuable secondary resource and a major source of environmental contamination. Rapid technological innovation and shortened product lifecycles have further accelerated e-waste accumulation [7]. Smartphones, laptops, and portable electronics are often discarded within only a few years despite remaining partially functional. Simultaneously, the expansion of electric vehicles, renewable energy storage systems, and advanced communication infrastructures has introduced new categories of electronic waste containing increasingly complex material compositions [8]. The environmental significance of e-waste pollution extends far beyond visible solid waste accumulation. Informal recycling operations, particularly in low- and middle-income countries, frequently employ primitive recovery techniques such as open burning, acid leaching, uncontrolled heating, and manual dismantling to extract valuable metals from discarded devices [9]. These practices release toxic gases, heavy metals, persistent organic pollutants, contaminated wastewater, and airborne particulate matter into surrounding environments. Elevated concentrations of lead, mercury, cadmium, copper, zinc, and brominated compounds have been detected in soil, sediments, groundwater, agricultural fields, and atmospheric particles near major recycling sites [10]. Long-term exposure to these contaminants has been associated with ecological degradation, microbial imbalance, reduced agricultural productivity, and chronic toxicity in nearby populations. Historically, research concerning e-waste pollution focused primarily on bulk heavy metals and persistent organic contaminants. However, increasing evidence suggests that e-waste processing and environmental degradation also generate nanoscale pollutants with physicochemical properties fundamentally different from their bulk counterparts [11,12]. The emergence of electronic waste-derived nanomaterials (EWNMs) has therefore introduced a new dimension of environmental and public health concern. EWNMs are nanoscale particles and complex nanostructures unintentionally generated during shredding, crushing, dismantling, thermal recovery, chemical extraction, and environmental weathering of electronic materials [13,14,15]. These materials may include metallic nanoparticles, metal oxides, carbonaceous nanostructures, nanoscale polymer fragments, semiconductor particles, and mixed nanocomposites originating from printed circuit boards, batteries, cables, and electronic display systems. Due to their extremely small size and high surface area-to-volume ratio, nanomaterials exhibit enhanced surface reactivity, catalytic activity, environmental mobility, and biological penetration potential compared with larger particulate contaminants [16,17]. Increasing evidence demonstrates that mechanical and thermal recycling activities can generate substantial quantities of airborne nanoscale particles. Crushing and shredding processes produce ultrafine metal-rich dust particles containing copper, chromium, nickel, tin, and rare earth elements. Thermal treatment methods including incineration and open burning facilitate the formation of metal oxide nanoparticles, soot-associated carbon nanoparticles, and secondary aerosol particles [18]. Similarly, chemical extraction procedures involving strong acids and oxidizing agents may generate nanoscale colloids and mixed nanocomposite materials through dissolution and reprecipitation mechanisms. Environmental weathering also contributes significantly to EWNM formation. Exposure of discarded electronic materials to sunlight, moisture, oxidative stress, microbial activity, and temperature fluctuations can gradually fragment bulk materials into nanoscale particles [19]. Polymeric insulation materials and brominated flame retardants may generate carbonaceous nanoparticles coated with toxic organic compounds, whereas degradation of lithium-ion batteries can release nanoscale lithium cobalt oxide, manganese oxide, and nickel-containing particles into environmental systems. Transformation of bulk electronic components into nanoscale pollutants substantially alters their environmental behavior. Reduced particle size increases atmospheric suspension time, dispersion potential, and interaction with biological and environmental interfaces [20]. EWNMs possess elevated surface energy and reactive functional groups that influence aggregation, dissolution, adsorption, oxidation, and interaction with natural organic matter. These properties enable nanoscale contaminants to undergo complex environmental transformations that determine their persistence, mobility, and toxicity. In aquatic environments, EWNMs may interact with dissolved ions, proteins, humic substances, and microbial exudates, altering particle stability and bioavailability [21]. In soil and sediment systems, nanoscale particles may adsorb onto mineral surfaces or migrate through pore water pathways, facilitating environmental transport and persistence. Their small size additionally enables uptake by microorganisms, algae, plants, and aquatic organisms, raising concerns regarding bioaccumulation and trophic transfer through food chains [22]. Recent toxicological investigations indicate that many EWNMs can induce reactive oxygen species (ROS) generation, oxidative stress, mitochondrial dysfunction, membrane damage, inflammatory signaling, and DNA damage in biological systems [23]. The physicochemical heterogeneity of EWNMs further complicates toxicity assessment because biological responses may vary according to particle composition, size, morphology, crystallinity, and surface chemistry. Certain metal-containing nanoparticles derived from e-waste have been associated with pulmonary toxicity, neurotoxicity, endocrine disruption, reproductive dysfunction, immunotoxicity, and genotoxicity in experimental studies. Human exposure to EWNMs may occur through inhalation, ingestion, dermal contact, and occupational exposure pathways [24]. Informal recycling workers represent one of the most vulnerable populations due to prolonged exposure to airborne ultrafine particles and contaminated environmental media during dismantling and material recovery activities. Residents living near recycling facilities may also experience chronic exposure through contaminated food, drinking water, soil, and atmospheric deposition [25]. Nanoscale pollutants possess the ability to penetrate deep into pulmonary tissues, cross epithelial barriers, enter systemic circulation, and accumulate in secondary organs including the liver, kidneys, brain, and reproductive tissues. Children, pregnant women, elderly individuals, and workers involved in recycling activities may experience greater vulnerability because of prolonged exposure duration and increased physiological sensitivity [26]. Nevertheless, despite increasing concern regarding EWNMs, epidemiological evidence describing long-term human health impacts remains limited. Current understanding of chronic exposure effects, environmental persistence, bioaccumulation, and ecosystem-level consequences remains insufficient, highlighting the urgent need for multidisciplinary investigations integrating nanotoxicology, environmental science, occupational health, and materials engineering. Electronic waste processing and environmental degradation generate a broad spectrum of nanoscale materials through mechanical fragmentation, thermal treatment, chemical corrosion, and long-term weathering. These materials are generally recognized as incidental nanomaterials because they are formed unintentionally rather than being deliberately engineered. Within this broader group, electronic waste-derived nanomaterials (EWNMs) represent nanoscale materials that originate specifically from discarded electronic products and their associated recycling or degradation processes. Depending on their formation pathway, EWNMs may include metallic and metal oxide nanoparticles, semiconductor-derived nanoparticles, carbonaceous nanomaterials, polymer-based Nano plastics, battery-derived nanomaterials, and mixed colloidal particles. Many airborne EWNMs also occur within the ultrafine particle (UFP; <100 nm) size range. However, particle size alone does not adequately describe their environmental behavior or potential health impacts because these materials exhibit considerable variability in chemical composition, surface chemistry, morphology, and transformation during environmental aging. Consequently, examining these particles from an e-waste source perspective provides a framework for understanding how their origin, physicochemical evolution, environmental transport, and exposure pathways collectively influence their environmental fate and toxicological responses.

### 1.2. Limitations of Current E-Waste Risk Assessment Approaches

Current environmental risk assessment frameworks for e-waste primarily focus on bulk contaminants such as heavy metals and persistent organic pollutants while largely overlooking nanoscale pollutants generated during recycling and environmental degradation processes. Traditional monitoring methods often fail to distinguish nanoscale particles from larger particulate matter despite substantial differences in mobility, surface reactivity, bioavailability, and toxicological behavior [27]. Analytical characterization of EWNMs remains technically challenging because nanoparticles undergo continuous environmental transformation through aggregation, dissolution, oxidation, corona formation, and surface functionalization. Variability in particle composition, morphology, and environmental interactions further complicates accurate exposure assessment and toxicity prediction. Existing regulatory guidelines also remain insufficient for addressing nanoscale e-waste contaminants because standardized protocols for sampling, environmental monitoring, toxicological evaluation, and risk characterization are still under development [28]. Consequently, significant uncertainties persist regarding environmental persistence, chronic exposure effects, ecological toxicity, and dose–response relationships associated with EWNMs. An additional limitation of current e-waste risk assessment frameworks is that they often evaluate nanoscale contaminants within broader categories such as incidental nanomaterials or ultrafine particles without adequately considering their source-specific characteristics. While these classifications provide valuable information on particle size, atmospheric transport, occupational exposure, and associated health outcomes, they do not fully capture the compositional complexity of nanoscale materials released from electronic waste. Electronic waste contains a heterogeneous mixture of metals, metal oxides, semiconductors, polymers, flame retardants, battery constituents, and rare earth elements that can generate chemically diverse nanoparticles through mechanical processing, thermal treatment, chemical extraction, and environmental weathering. As these particles undergo continuous physicochemical transformation after their release, their environmental behavior and biological interactions may differ substantially from those of conventional ultrafine particles originating from combustion or urban air pollution. Therefore, future risk assessment strategies should integrate source-specific material composition, transformation pathways, environmental aging, and multimedia exposure routes alongside established particle-based approaches to achieve a more comprehensive evaluation of the environmental and public health risks associated with e-waste-derived nanomaterials.

### 1.3. Scope, Novelty, and Objectives of the Review

Although recent review articles have significantly advanced the understanding of electronic waste management, environmental contamination, recycling technologies, and nanomaterial-related health effects, their primary emphasis has remained on bulk e-waste pollutants, resource recovery, engineered nanomaterials, or general environmental sustainability rather than on nanoscale contaminants generated from electronic waste itself [26,27,28,29,30,31]. Existing reviews generally address these topics independently and provide only limited coverage of the formation mechanisms, physicochemical evolution, environmental transformation, analytical characterization, biological interactions, and toxicological implications of electronic waste-derived nanomaterials (EWNMs). Consequently, a comprehensive source-oriented assessment integrating these interconnected aspects remains lacking. To address this knowledge gap, the present review provides a comprehensive synthesis of the current understanding of EWNMs, encompassing their generation during electronic waste recycling and environmental weathering, physicochemical characteristics, environmental release, transport and transformation, exposure pathways, toxicological mechanisms, analytical characterization, risk assessment, mitigation strategies, and techno-economic considerations. By integrating evidence from environmental science, nanotoxicology, materials science, occupational health, and waste management, this review establishes a multidisciplinary framework for evaluating the environmental and public health significance of EWNMs while identifying critical research priorities for their sustainable management.

The novelty and major objectives of this review are summarized as follows:

1. To provide a comprehensive overview of the global expansion of electronic waste generation and the increasing environmental burden associated with modern electronic consumption patterns.
2. To discuss the mechanisms involved in the formation of electronic waste-derived nanomaterials during dismantling, shredding, thermal recovery, chemical extraction, and environmental weathering processes.
3. To examine the physicochemical characteristics of EWNMs, including particle size distribution, morphology, surface chemistry, aggregation behavior, and environmental transformation pathways.
4. To evaluate the environmental release, transport, persistence, and fate of EWNMs in air, soil, water, and sediment systems.
5. To critically analyze human exposure pathways, including inhalation, ingestion, dermal exposure, and occupational exposure in informal and industrial recycling settings.
6. To summarize current evidence regarding the toxicological effects of EWNMs, including oxidative stress, reactive oxygen species generation, inflammation, neurotoxicity, genotoxicity, reproductive toxicity, endocrine disruption, and immunotoxicity.
7. To assess the ecological impacts of EWNMs on microorganisms, plants, aquatic organisms, food chains, and ecosystem stability.
8. To discuss recent advances in analytical characterization and detection techniques used for identifying and monitoring nanoscale pollutants derived from electronic waste.
9. To identify major limitations in current environmental risk assessment models and regulatory frameworks associated with nanoscale e-waste contaminants.
10. To explore emerging sustainable mitigation strategies, including green recycling technologies, circular economy approaches, safer material design, nanomaterial recovery systems, environmentally responsible waste management practices, and techno-economic considerations.
11. To highlight critical research gaps and propose future directions for interdisciplinary investigations related to EWNM toxicity, environmental monitoring, exposure assessment, engineering controls, and regulatory policy development.

## 2. Global Generation and Composition of Electronic Waste

### 2.1. Global E-Waste Production Trends

**Figure 1:**
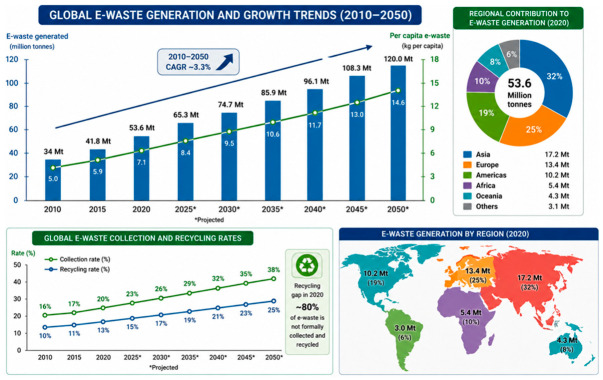
Global Electronic Waste Generation and Growth Trends (2010–2050). Global trends in electronic waste generation, regional contributions, collection and recycling rates, and projected future growth of e-waste under increasing technological consumption and shortened device lifecycles. Graph prepared from Global E-waste Monitor reports (2017, 2020, and 2024) and international e-waste statistics open databases Ref. [30].

Electronic waste generation has increased dramatically over the past few decades due to rapid technological advancement, expanding digital infrastructure, rising consumer demand, and shortened product lifecycles. Increasing urbanization and economic development have accelerated the adoption of electronic devices across residential, commercial, industrial, healthcare, and transportation sectors. Simultaneously, declining repair ability, planned obsolescence, and continuous software upgrades have contributed to the rapid disposal of electronic products before the end of their functional lifespan [29]. As a result, e-waste has emerged as one of the fastest-growing waste streams globally, creating significant environmental, economic, and public health concerns. Recent global assessments indicate that annual electronic waste production has exceeded 60 million metric tons, with projections suggesting continuous growth over the coming decades [30]. The increasing penetration of smartphones, laptops, televisions, wearable electronics, cloud computing infrastructure, and smart appliances has significantly contributed to rising e-waste volumes worldwide. According to The Global E-waste Monitor 2020, only 20.0% of the 53.6 million metric tons of electronic waste generated globally in 2019 was formally collected and recycled, while the remaining waste was unmanaged, improperly processed, or entered informal recycling stream. Despite the enormous quantity of discarded electronic materials, only a limited fraction is formally collected and recycled through environmentally regulated systems [31]. Large amounts of electronic waste are either stored in households, discarded in landfills, exported to developing countries, or processed using informal recycling practices. Per capita e-waste generation has also increased substantially in both developed and emerging economies. High-income countries generally generate larger quantities of electronic waste per individual due to greater purchasing capacity and faster technological replacement cycles [32]. However, rapidly industrializing nations are experiencing accelerated increases in e-waste generation because of expanding digital accessibility and rising electronic consumption. Figure 1 shows worldwide generation of electronic waste from 2010 to 2050, illustrating historical growth, projected future increases, regional distribution patterns, and collection and recycling rates. Also the figure highlights the expanding global e-waste burden and the growing gap between waste generation and formal recycling capacity [30].

The geographical distribution of electronic waste generation varies significantly across different regions [33]. Asia represents the largest contributor to global e-waste production due to its large population, rapid industrial growth, and extensive electronics manufacturing activities. Countries such as China, India, Japan, and South Korea contribute substantially to regional e-waste accumulation. Europe and North America also generate considerable quantities of electronic waste because of high electronic consumption rates and frequent product replacement cycles [34]. In contrast, many African and South American countries generate comparatively lower quantities of e-waste but frequently receive imported electronic waste from developed nations for recycling and disposal purposes. Informal recycling hubs in countries such as Ghana, Nigeria, India, Pakistan, and China have become major centers for manual dismantling and crude material recovery. These activities often occur under inadequate environmental regulations and occupational safety conditions, increasing the risk of environmental contamination and human exposure to hazardous pollutants. Future projections indicate that global e-waste generation will continue increasing due to technological innovation, population growth, urban expansion, and digital transformation [30]. The growing demand for artificial intelligence systems, electric vehicles, renewable energy technologies, data storage infrastructure, and internet-connected devices is expected to accelerate electronic consumption worldwide. Emerging technologies including 5G communication systems, smart cities, wearable health devices, and advanced battery systems may further contribute to the diversification and complexity of future electronic waste streams. In addition, the transition toward renewable energy technologies has introduced new forms of electronic waste associated with photovoltaic panels, lithium-ion batteries, and energy storage systems [8]. These components contain complex mixtures of metals, polymers, and critical raw materials that may generate additional environmental challenges during disposal and recycling.

### 2.2. Major Sources and Categories of Electronic Waste

**Figure 2:**
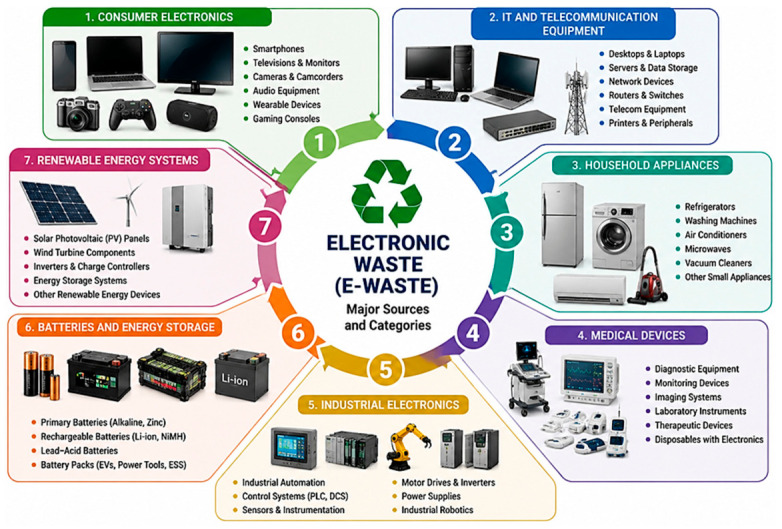
Major Sources and Categories of Electronic Waste: Schematic classification of electronic waste according to major product groups and end-use sectors contributing to global e-waste generation.

Electronic waste originates from a broad range of consumer, commercial, medical, industrial, and energy-related electronic products. The diversity of electronic devices contributes to substantial variability in material composition, toxic constituents, recycling complexity, and environmental impact. Consumer electronics represent one of the largest categories of electronic waste worldwide [35]. This category includes smartphones, televisions, laptops, tablets, cameras, audio systems, gaming devices, and wearable electronics. Rapid technological innovation and consumer demand for upgraded features frequently shorten the lifespan of these products, leading to increased disposal rates. Modern consumer electronics contain complex assemblies of printed circuit boards, semiconductor chips, batteries, display panels, and polymeric casings. These materials often contain hazardous substances including lead, brominated flame retardants, cadmium, and rare earth elements [36]. Information technology (IT) equipment including servers, desktop computers, networking systems, storage devices, printers, and telecommunication infrastructure contributes significantly to global e-waste generation. Rapid digitalization and expansion of cloud computing services have accelerated the replacement of outdated IT systems across commercial and institutional sectors. Data centers and telecommunication facilities generate substantial quantities of obsolete electronic components containing metals, semiconductors, cooling materials, and electronic circuitry [37]. Disposal of these materials without proper recycling may contribute to heavy metal release and nanoscale pollutant generation. Large and small household appliances constitute another major category of electronic waste [38]. Refrigerators, air conditioners, washing machines, microwave ovens, vacuum cleaners, and kitchen appliances contain electrical wiring, compressors, insulation materials, plastics, and metal alloys. Many of these devices also contain refrigerants, flame retardants, and hazardous additives capable of causing environmental contamination during improper disposal. The increasing adoption of smart household technologies and internet-connected appliances is expected to further increase household-related electronic waste generation in the coming years. Medical electronics including diagnostic instruments, monitoring systems, imaging devices, laboratory equipment, and implantable technologies represent an emerging source of specialized electronic waste [38]. These devices often contain highly complex electronic assemblies, sensors, batteries, and heavy metals requiring specialized recycling procedures. Industrial electronics used in manufacturing systems, robotics, automation technologies, and energy infrastructure also contribute to increasing volumes of electronic waste. Disposal and recycling of industrial electronic equipment may release hazardous substances and ultrafine particulate contaminants into occupational and environmental settings. The rapid expansion of renewable energy technologies has introduced new categories of electronic waste associated with photovoltaic panels, wind energy systems, and lithium-ion batteries [8]. Solar panels contain silicon wafers, silver, cadmium telluride, lead, and polymeric encapsulation materials, while lithium-ion batteries contain lithium, cobalt, nickel, manganese, and electrolyte compounds. Improper disposal or recycling of these materials may contribute to environmental contamination and generation of nanoscale metal-containing pollutants. The anticipated growth of electric vehicles and renewable energy storage systems is expected to significantly increase future battery-related waste streams. A schematic overview is presented in Figure 2.

### 2.3. Material Composition of Electronic Waste

Electronic waste contains highly diverse material compositions that influence recycling efficiency, environmental toxicity, and nanoparticle generation potential. Metals constitute a major fraction of electronic waste and include both valuable and toxic elements [39]. Commonly detected metals include copper, aluminum, iron, gold, silver, palladium, platinum, lithium, cobalt, chromium, mercury, cadmium, and lead. Printed circuit boards and batteries represent particularly metal-rich components. Mechanical fragmentation, thermal treatment, and chemical extraction of metal-containing electronic materials may generate ultrafine metal particles and nanoscale metal oxides capable of environmental transport and biological interaction [40]. Plastic materials are extensively used in electronic casings, insulation systems, cables, and structural components. Common polymers include polyvinyl chloride, polyethylene, polypropylene, acrylonitrile butadiene styrene, and polycarbonate materials.

**Figure 3:**
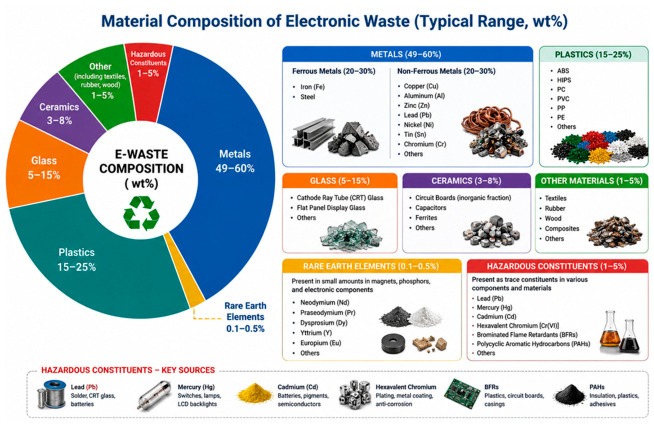
Schematic representation of the major material constituents of electronic waste, illustrating the relative distribution of metallic components, polymeric materials, glass, ceramics, rare earth elements, and hazardous substances. The figure highlights the complex and heterogeneous composition of e-waste, emphasizing both its resource recovery potential and its significance as a source of environmental contamination.

Many plastics contain flame retardants, plasticizers, stabilizers, and additives that may release toxic compounds during thermal degradation or environmental weathering. The overall process is illustrated in Figure 3. Combustion and degradation of polymeric electronic materials can also generate carbonaceous nanoparticles and toxic gaseous emissions [41]. Modern electronic devices increasingly rely on rare earth elements and critical raw materials for advanced technological applications. Neodymium, lanthanum, cerium, dysprosium, indium, gallium, and tantalum are commonly used in magnets, semiconductors, displays, batteries, and renewable energy systems. Recovery of these materials is economically important but technologically challenging because of their low concentrations and complex associations with other materials. Electronic devices contain various hazardous additives including brominated flame retardants, chlorinated compounds, polychlorinated biphenyls, phthalates, and persistent organic pollutants [42]. These substances may leach into environmental systems during landfill disposal, open burning, and recycling operations. Thermal treatment and environmental degradation of such compounds may additionally generate toxic secondary pollutants and nanoscale carbonaceous particles capable of long-range environmental transport.

### 2.4. Informal Recycling Practices in Developing Countries

Informal recycling sectors play a major role in electronic waste processing in many developing countries. These activities are primarily driven by economic incentives associated with recovery of valuable metals from discarded electronic devices. Open burning is widely used for recovering copper and other valuable metals from cables and circuit boards [43]. Primitive smelting techniques involving uncontrolled heating are also commonly employed. These practices release toxic gases, soot particles, heavy metals, and nanoscale particulate matter into surrounding environments. Acid leaching processes using nitric acid, hydrochloric acid, sulfuric acid, and cyanide-containing solutions are frequently used for metal extraction in informal recycling facilities. Improper handling and disposal of these chemicals may contaminate nearby water systems and generate nanoscale metal-containing colloids. Workers in informal recycling sectors are exposed to hazardous chemicals, airborne particulate matter, toxic fumes, and contaminated wastewater under poorly regulated conditions [44,45]. Prolonged exposure may increase the risk of respiratory disorders, neurological damage, oxidative stress, reproductive toxicity, and chronic inflammatory diseases.

### 2.5. Environmental Burden Associated with Improper E-Waste Management

Improper management of electronic waste contributes significantly to environmental pollution, ecosystem degradation, and human health risks. Landfilling, uncontrolled dumping, open burning, and primitive recycling release toxic metals, persistent organic pollutants, microplastics, and nanoscale contaminants into air, water, and soil systems. Environmental contamination associated with e-waste recycling has been linked to biodiversity loss, soil infertility, aquatic toxicity, food chain contamination, and atmospheric pollution [46]. In addition, nanoscale pollutants generated during recycling and environmental weathering may exhibit enhanced mobility, persistence, and biological reactivity compared with conventional contaminants. The growing complexity of electronic materials and increasing global e-waste generation highlight the urgent need for sustainable recycling technologies, circular economy approaches, stricter environmental regulations, and improved international cooperation to minimize future environmental burden and public health risks. For a better understanding, Table 1 represents the global e-waste generation continues to increase across all major regions, with Asia contributing the largest overall volume and Europe exhibiting the highest collection efficiency. Despite advances in recycling technologies, formal recycling rates remain insufficient, particularly in developing regions where informal recycling practices dominate. Continued growth in electronic consumption, renewable energy technologies, and battery usage is expected to further increase e-waste generation and the potential environmental release of electronic waste-derived nanomaterials (EWNMs).

## 3. Formation and Transformation of E-Waste-Derived Nanomaterials

### 3.1. Classification of Electronic Waste-Derived Nanomaterials

Electronic waste-derived nanomaterials (EWNMs) comprise a chemically diverse group of incidental nanomaterials generated during electronic waste recycling, disposal, and environmental weathering. Their composition is determined by the type of electronic component, processing conditions, and transformation pathways. Unlike engineered nanomaterials, EWNMs exhibit considerable variability in size, morphology, crystallinity, surface chemistry, and environmental reactivity because they originate from heterogeneous waste streams. For clarity, EWNMs can be systematically classified according to their dominant chemical composition and source materials into metallic nanoparticles, metal oxide nanoparticles, carbon-based nanomaterials, semiconductor nanomaterials, polymer-derived nanoplastics, and hybrid nanomaterials, as shown in Table 2. This classification facilitates a better understanding of their formation mechanisms, physicochemical characteristics, environmental behavior, and potential toxicological effects.

### 3.2. Overview of Nanomaterial Formation from E-Waste

Electronic waste serves as an important source of unintentionally generated nanomaterials formed through mechanical processing, thermal treatment, chemical corrosion, and long-term environmental weathering. During recycling, disposal, storage, and environmental exposure, bulk electronic components progressively degrade and fragment, producing nanoscale particles with diverse physicochemical characteristics [40]. Unlike engineered nanomaterials that are intentionally synthesized, electronic waste-derived nanomaterials (EWNMs) are generated incidentally through complex transformation pathways involving mechanical stress, thermal decomposition, oxidation, photodegradation, and biological activity. Consequently, EWNMs comprise a heterogeneous mixture of metallic and metal oxide nanoparticles, carbonaceous nanomaterials, semiconductor nanoparticles, polymer-derived nanoplastics, and hybrid nanocomposites, with compositions that vary according to the electronic waste source, processing conditions, and environmental aging [47]. The formation of EWNMs during thermal recycling processes is governed by interconnected physicochemical and reaction engineering phenomena. During pyrolysis, combustion, or uncontrolled open burning, elevated temperatures promote polymer decomposition and metal volatilization, generating gaseous species that subsequently undergo homogeneous or heterogeneous nucleation to form primary nanoparticles. These particles continue to grow through vapor condensation, surface reactions, coagulation, and aggregation, resulting in changes in particle size distribution and morphology. The extent of these processes is strongly influenced by operating conditions, including temperature, heating rate, residence time, oxygen availability, and cooling rate. Lower temperatures generally favor the release of condensable organic compounds and incomplete decomposition products, whereas higher temperatures enhance gas-to-particle conversion and the formation of metal and metal oxide nanoparticles. Consequently, the physicochemical properties and environmental behavior of EWNMs are governed by the combined effects of reaction kinetics, thermodynamic equilibrium, and post-formation transformations during thermal processing and subsequent environmental aging.

### 3.3. Mechanical Fragmentation Processes

Mechanical processing represents one of the primary pathways for the generation of electronic waste-derived nanomaterials (EWNMs) during recycling and material recovery operations. Manual dismantling, component separation, crushing, shredding, grinding, and milling subject electronic components to repeated impact, compression, abrasion, and shear forces, progressively reducing bulk materials into micro- and nanoscale particles [48]. Printed circuit boards, batteries, cables, display panels, electronic connectors, and polymeric housings are particularly susceptible to fragmentation because they consist of heterogeneous materials with different mechanical properties. Manual dismantling generally produces localized particle emissions during cutting, drilling, and component separation, whereas subsequent crushing and shredding substantially increase nanoparticle generation by creating fresh fracture surfaces and accelerating fragmentation of brittle materials [49]. Mechanical fragmentation produces complex mixtures of metallic nanoparticles, metal oxide nanoparticles, ceramic fragments, glass particles, semiconductor materials, and polymer-derived nanoplastics. Airborne particles generated during shredding operations have been reported to contain elevated concentrations of copper, aluminum, chromium, nickel, lead, tin, and rare earth elements, with a substantial fraction occurring within the ultrafine and nanoscale size range [49]. Their small size and large specific surface area enhance atmospheric transport, environmental dispersion, and inhalation exposure, thereby increasing potential human and ecological risks. Abrasion is another important mechanism contributing to nanoparticle generation during electronic waste handling and recycling [50]. Continuous friction between electronic components, conveyor systems, cutting tools, and processing equipment gradually erodes material surfaces, releasing nanoscale debris with irregular morphology and highly reactive surfaces. These particles readily interact with surrounding contaminants and may undergo aggregation, oxidation, or surface modification after environmental release, altering their mobility, persistence, and biological interactions. Electronic devices also contain substantial quantities of polymeric materials used in casings, cable insulation, printed circuit board laminates, and composite components [51]. Mechanical processing of these materials generates nanoscale polymer fragments and nanoplastics through brittle fracture, abrasion, and progressive degradation. These particles frequently retain brominated flame retardants, plasticizers, stabilizers, pigments, and other additives inherited from the parent material, which can modify their environmental behavior and toxicological properties [52]. Consequently, polymer-derived nanoplastics may serve as carriers for hazardous chemicals, facilitating their transport and bioavailability in environmental systems.

The characteristics and abundance of mechanically generated EWNMs are strongly influenced by waste composition, equipment design, operating conditions, particle residence time, and dust control efficiency. The quantity and characteristics of mechanically generated EWNMs are influenced by the composition of electronic waste, processing equipment, operating conditions, and particle fragmentation mechanisms, resulting in considerable variability in particle size, morphology, and chemical composition.

### 3.4. Thermal Degradation and Combustion Processes

Thermal treatment processes are major contributors to EWNM formation. High-temperature conditions alter the chemical composition and physical structure of electronic materials, promoting the generation of complex nanoparticle mixtures. Open burning remains a widespread informal recycling practice in many developing countries where valuable metals are recovered from electronic waste [53]. During combustion, polymeric materials, insulation coatings, and circuit board substrates undergo thermal decomposition, producing large quantities of ultrafine particulate matter. Combustion-generated particles commonly contain heavy metals, metal oxides, soot, polycyclic aromatic hydrocarbons, and brominated compounds. Due to incomplete combustion conditions, nanoparticles generated during open burning often exhibit highly reactive surfaces capable of adsorbing toxic organic contaminants. Pyrolysis technologies have gained attention as potential alternatives to uncontrolled burning for resource recovery from electronic waste [54]. Pyrolysis involves thermal decomposition under oxygen-limited conditions, facilitating recovery of valuable metals and energy-rich products. Although pyrolysis reduces certain pollutant emissions compared with open burning, nanoparticle formation still occurs during thermal decomposition processes. Metal vapors generated at elevated temperatures may subsequently condense to form nanoscale particles, while decomposition of polymeric materials produces carbonaceous nanostructures and secondary aerosols. High-temperature processing promotes oxidation reactions that convert metallic components into metal oxide nanoparticles [55]. Copper oxide, zinc oxide, iron oxide, nickel oxide, cobalt oxide, and manganese oxide nanoparticles have been identified in emissions associated with thermal recycling operations. The formation process generally involves volatilization of metals followed by oxidation and condensation within cooling gas streams. Resulting nanoparticles often possess high surface reactivity and enhanced biological activity compared with bulk metal oxides. Combustion and thermal decomposition of polymers produce significant quantities of carbonaceous nanoparticles [56]. These particles include soot nanoparticles, amorphous carbon structures, carbon nanospheres, and graphitic fragments. Their formation is influenced by combustion temperature, oxygen availability, and polymer composition. Carbonaceous nanoparticles frequently adsorb heavy metals and organic pollutants onto their surfaces, generating complex hybrid contaminants capable of long-range environmental transport.

### 3.5. Chemical Leaching and Corrosion Mechanisms

Chemical transformation processes play a crucial role in the generation of nanoscale contaminants from electronic waste. Corrosion, dissolution, and precipitation reactions continuously modify electronic materials during recycling and environmental exposure [57]. Acid leaching is widely employed for recovering valuable metals from electronic waste, particularly in informal recycling sectors. Nitric acid, hydrochloric acid, sulfuric acid, and mixed acid solutions are commonly used to dissolve metallic components. [58]. Environmental exposure and recycling processes frequently promote oxidation and dissolution of metallic components. Moisture, oxygen, acidic conditions, and electrochemical reactions accelerate corrosion of printed circuit boards, batteries, and metallic connectors. Dissolution releases metal ions into surrounding media, while oxidation modifies surface chemistry and promotes formation of nanoscale corrosion products. Such reactions are particularly important for copper, iron, zinc, silver, and battery-associated metals. Following dissolution, dissolved metal ions may undergo reprecipitation through changes in pH, redox conditions, ionic strength, and chemical composition of surrounding media [59]. These processes facilitate nucleation and growth of metal-containing nanoparticles. Reprecipitated particles frequently exhibit distinct physicochemical properties compared with their parent materials. The resulting nanoparticles may remain suspended as colloids or become incorporated into environmental matrices such as sediments and soils.

### 3.6. Environmental Weathering and Aging Processes

Electronic waste continues to undergo transformation long after disposal. Environmental weathering processes progressively alter material structure, composition, and surface characteristics, promoting continuous nanoparticle generation. Solar ultraviolet (UV) radiation is a major driver of degradation in polymeric and composite electronic materials [60]. A summary of the key characteristics is provided in Table 3. Prolonged UV exposure breaks chemical bonds, weakens material integrity, and promotes surface cracking. Photodegradation increases susceptibility to fragmentation and facilitates release of nanoscale particles from electronic casings, cable coatings, and composite materials. UV-induced reactions may additionally alter surface chemistry and increase particle reactivity. Environmental moisture and oxygen continuously interact with discarded electronic materials, initiating hydrolytic and oxidative degradation processes [61]. Hydrolysis contributes to breakdown of polymers and composite materials, while oxidation accelerates corrosion of metallic surfaces. These weathering mechanisms progressively generate nanoscale fragments and secondary particles that may be transported through environmental systems. Weathering products often differ substantially from the original materials in terms of composition, morphology, and reactivity. Microorganisms can influence transformation of electronic waste through biodegradation, bioleaching, and bio-mineralization processes [62]. Bacteria and fungi capable of colonizing electronic waste surfaces produce enzymes, organic acids, and extracellular polymers that facilitate material degradation. Microbial activity may accelerate metal dissolution, alter redox conditions, and promote formation of nanoscale mineral phases. Bioleaching microorganisms are particularly important in the mobilization and transformation of metal-containing electronic waste components. Environmental aging modifies nanoparticle surfaces through adsorption of natural organic matter, inorganic ions, proteins, and microbial metabolites [63]. These interactions influence particle stability, aggregation behavior, dissolution rates, and biological interactions. Formation of environmentally derived surface coatings, often referred to as eco-coronas, can significantly alter nanoparticle transport and toxicity. Consequently, aged EWNMs may exhibit environmental behaviors that differ markedly from freshly generated particles.

### 3.7. Hybrid and Secondary Nanomaterial Formation

**Figure 4:**
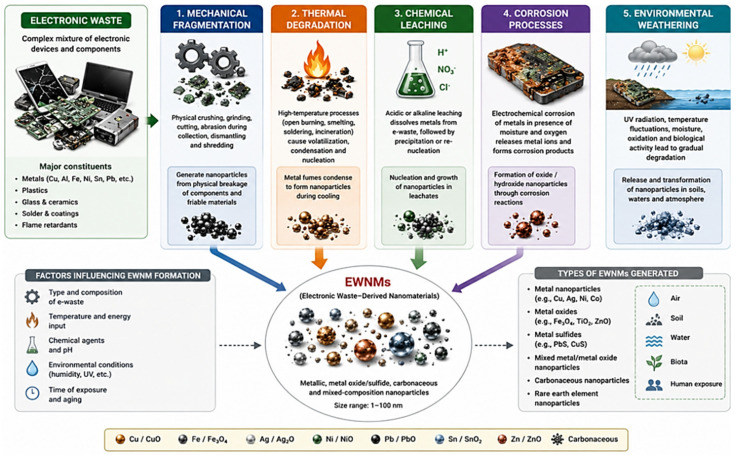
Schematic presentation of formation pathways of electronic waste-derived nanomaterials (EWNMs): Generation of nanoparticles through mechanical fragmentation, thermal degradation, chemical leaching, corrosion, and environmental weathering.

The complexity of electronic waste frequently leads to the formation of hybrid and secondary nanomaterials through interactions among metals, polymers, carbonaceous materials, and environmental constituents. During recycling and environmental exposure, primary nanoparticles rarely remain chemically unchanged. Instead, they undergo continuous transformation through aggregation, adsorption, dissolution, and re-precipitation processes. Hybrid nanomaterials may consist of metal-metal oxide composites, carbon-metal mixtures, polymer-coated nanoparticles, or environmentally transformed colloidal structures [69]. These materials often possess physicochemical properties distinct from those of their original components. The major pathways involved are illustrated in Figure 4. Adsorption of heavy metals, persistent organic pollutants, and natural organic matter onto nanoparticle surfaces can further increase structural complexity and environmental persistence. Secondary nanoparticle formation is particularly important because it influences mobility, bioavailability, and toxicity in environmental systems [66]. Through ongoing transformation processes, EWNMs may evolve into highly heterogeneous contaminant mixtures capable of interacting with biological systems through multiple mechanisms. Understanding these dynamic transformation pathways is therefore essential for accurate environmental risk assessment and development of effective mitigation strategies.

## 4. Environmental Release and Transport Mechanisms

The environmental release and subsequent transport of electronic waste-derived nanomaterials (EWNMs) play a critical role in determining their environmental distribution, persistence, exposure potential, and ecological impacts. Once released during electronic waste recycling, disposal, or environmental weathering, EWNMs undergo continuous transport and transformation across air, water, soil, sediment, and groundwater systems. Their environmental fate is governed by interactions between intrinsic particle characteristics, including size, morphology, surface charge, and chemical composition, and external factors such as pH, ionic strength, natural organic matter, temperature, and hydrodynamic conditions. These interactions influence particle mobility, aggregation, sedimentation, dissolution kinetics, surface transformations, and ultimately their environmental persistence and biological availability.

### 4.1. Environmental Entry Pathways of EWNMs

**Figure 5:**
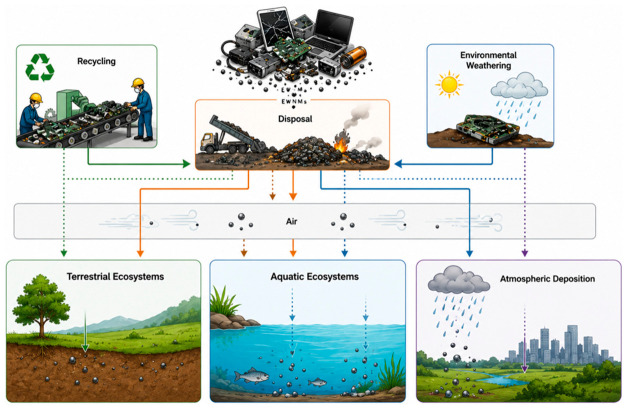
Conceptual overview of the major pathways facilitating the entry of EWNMs into air, soil, sediment, and water systems following recycling operations, waste disposal, and environmental aging of electronic materials.

Electronic waste-derived nanomaterials (EWNMs) are introduced into environmental systems through multiple pathways associated with recycling, disposal, storage, transportation, and environmental weathering of discarded electronic products. Unlike conventional pollutants that may remain localized near contamination sources, nanoscale materials possess unique physicochemical characteristics that facilitate mobility across environmental compartments. As a result, EWNMs can be released into atmospheric, terrestrial, and aquatic environments, where they undergo complex transport and transformation processes. Major entry pathways include emissions from mechanical recycling operations, thermal recovery processes, open burning activities, wastewater discharge from recycling facilities, landfill leachates, atmospheric deposition, and environmental degradation of improperly discarded electronic materials [70] see in Figure 5. Once released, EWNMs may interact with natural organic matter, mineral surfaces, dissolved ions, and biological systems, influencing their distribution, persistence, and ecological impacts. Understanding these release pathways is essential for evaluating environmental exposure and risk associated with nanoscale contaminants derived from electronic waste.

### 4.2. Atmospheric Release and Airborne Transport

The atmosphere represents one of the primary environmental compartments receiving EWNMs generated during electronic waste processing. Airborne nanoparticles are of particular concern because they can be transported over long distances and serve as an important route of human and ecological exposure. Mechanical dismantling, shredding, crushing, grinding, and material separation operations generate substantial quantities of airborne particulate matter enriched with nanoscale contaminants [71]. During these processes, physical fragmentation of electronic components releases metallic nanoparticles, metal oxide particles, polymer fragments, and carbonaceous nanomaterials into surrounding air. Thermal recovery operations and informal recycling activities further increase atmospheric emissions. Open burning of cables, circuit boards, and plastic components releases complex mixtures of ultrafine particles containing heavy metals, soot, and organic pollutants. Workers operating in poorly ventilated facilities often experience direct exposure to these emissions. Due to their small size and low settling velocity, nanoscale particles remain suspended in air for extended periods compared with larger particles [68]. Atmospheric turbulence, wind currents, temperature gradients, and human activities facilitate rapid dispersion of EWNMs from emission sources. Particle size, morphology, density, and surface characteristics significantly influence dispersion behavior. Ultrafine particles with diameters below 100 nm exhibit particularly high mobility and can penetrate indoor and outdoor environments with relative ease. Such properties increase the spatial extent of contamination around recycling facilities and disposal sites. Long-range atmospheric transport can distribute EWNMs far beyond their original emission sources [72]. Once suspended in the atmosphere, nanoparticles may be transported over regional and even continental scales through prevailing wind systems. During transport, particles undergo aging processes including oxidation, aggregation, condensation, and interaction with atmospheric pollutants. These transformations may alter particle composition, reactivity, and toxicity. Atmospheric deposition subsequently transfers airborne EWNMs to soil, vegetation, freshwater systems, and marine environments, thereby linking atmospheric contamination with other environmental compartments. The concentrations and particle size distributions of EWNMs can vary considerably among environmental matrices because they are influenced by electronic waste composition, recycling practices, environmental conditions, and analytical methodologies. Other published studies consistently demonstrate the occurrence of nanoscale contaminants in air, soil, water, sediments, and landfill leachates surrounding electronic waste recycling and disposal sites. In Table 4, an overview of the current evidence regarding the environmental occurrence of EWNMs. Atmospheric transport is further influenced by factors that determine airborne residence time, long-range dispersion, and respiratory exposure potential.

### 4.3. Soil Contamination and Terrestrial Distribution

Soil systems act as major environmental sinks for EWNMs due to direct deposition, disposal activities, and atmospheric fallout. Accumulation of nanoscale contaminants in terrestrial environments may influence soil quality, microbial communities, plant health, and food safety. Deposition of EWNMs occurs through atmospheric settling, precipitation events, landfill leakage, and direct release from recycling operations [64]. Nanoparticles emitted into the atmosphere eventually settle onto soil surfaces through dry and wet deposition processes. In addition, disposal of electronic waste in uncontrolled landfills facilitates direct release of nanoparticles and nanoparticle precursors into surrounding terrestrial environments. Recycling residues and contaminated wastewater may further contribute to localized soil contamination near processing facilities. Once deposited in soils, EWNMs interact extensively with mineral particles, clay surfaces, dissolved organic matter, and microbial exudates [67]. Soil organic matter plays a particularly important role in determining nanoparticle mobility and bioavailability. The summary of the soil deposition mechanism illustrated in Table 5. Adsorption of humic substances and organic molecules onto nanoparticle surfaces may stabilize colloidal suspensions and enhance transport through soil pore networks. Conversely, aggregation and attachment to soil particles can reduce mobility and promote long-term accumulation within terrestrial ecosystems. Growing evidence suggests that certain EWNMs can be absorbed by plant root systems and translocated to above-ground tissues. Nanoparticle uptake depends on particle size, surface charge, chemical composition, plant species, and soil characteristics. Following root absorption, nanoparticles may accumulate in stems, leaves, fruits, and edible tissues. Such processes raise concerns regarding food chain contamination and potential transfer of nanoscale pollutants to humans and animals through agricultural products. Within soils, mineral composition, pH, organic matter content, and pore-water chemistry regulate nanoparticle retention, vertical migration, and potential uptake by plants and soil microorganisms.

### 4.4. Aquatic Release and Waterborne Transport

Aquatic ecosystems represent important recipients of EWNMs originating from recycling activities, landfill disposal, atmospheric deposition, and surface runoff. Waterborne transport contributes significantly to the environmental distribution of nanoscale contaminants. Wastewater generated during dismantling, washing, metal recovery, and chemical extraction processes frequently contains suspended nanoparticles and dissolved metal species [65]. In regions lacking adequate wastewater treatment infrastructure, contaminated effluents may be discharged directly into rivers, lakes, wetlands, and coastal environments. These discharges introduce metallic nanoparticles, metal oxides, polymer fragments, and complex nanocomposites into aquatic ecosystems, potentially affecting water quality and aquatic biodiversity. Rainfall events facilitate mobilization of EWNMs from disposal sites, recycling facilities, and contaminated soils through surface runoff and leaching processes [75]. Nanoparticles transported by runoff can enter streams, rivers, reservoirs, and groundwater systems. Landfill leachates generated during weathering of discarded electronic materials may also serve as important sources of nanoparticle contamination. Dissolved metal ions and nanoparticle precursors present in leachates can undergo secondary transformations during transport through aquatic environments. Once introduced into aquatic systems, EWNMs undergo dynamic processes that influence their distribution and persistence [79]. Aggregation is one of the most important mechanisms controlling nanoparticle transport. Interactions with dissolved salts, natural organic matter, and suspended particles often promote formation of larger aggregates. Aggregation may enhance sedimentation and transfer nanoparticles from the water column to bottom sediments. However, certain environmental conditions may stabilize nanoparticle suspensions, allowing prolonged transport through aquatic systems. In aquatic environments, aggregation, dissolution, adsorption onto suspended particles, and interactions with natural organic matter strongly influence nanoparticle mobility, stability, and bioavailability.

### 4.5. Bioavailability and Environmental Persistence

The environmental risk associated with EWNMs depends not only on their concentration but also on their bioavailability and persistence within natural systems. These factors are strongly influenced by physicochemical transformations occurring after environmental release. Surface chemistry plays a central role in determining nanoparticle behavior in environmental systems [74]. Surface charge, functional groups, adsorbed contaminants, and environmental coatings influence aggregation, dissolution, and biological interactions. Colloidal stability affects the ability of nanoparticles to remain suspended and mobile within air, water, and soil environments. Stable nanoparticle suspensions generally exhibit greater environmental mobility and increased opportunities for biological exposure. EWNMs undergo continuous transformation after environmental release [73]. Dissolution processes may release metal ions into surrounding media, while oxidation, reduction, sulfidation, and surface functionalization alter particle composition and reactivity. Environmental transformations can either increase or decrease toxicity depending on the specific conditions involved. Consequently, accurate risk assessment requires consideration of both primary particles and their transformation products. The small size and high reactivity of EWNMs facilitate uptake by microorganisms, algae, plants, invertebrates, and vertebrates [21]. Once incorporated into biological systems, nanoparticles may accumulate within tissues and potentially transfer between trophic levels. Evidence from laboratory and field studies indicates that nanoscale contaminants can move through aquatic and terrestrial food webs, resulting in biomagnification concerns for higher organisms. Although the extent of trophic transfer remains under investigation, existing findings highlight the importance of considering food chain exposure in environmental risk assessments.

### 4.6. Environmental Fate Modeling of EWNMs

Environmental fate modeling has emerged as an important tool for predicting the distribution, transport, transformation, and long-term behavior of EWNMs in complex environmental systems. Such models integrate information regarding particle properties, emission rates, environmental conditions, and transformation processes to estimate environmental concentrations and exposure scenarios. Current modeling approaches incorporate atmospheric transport, hydrological movement, sediment interactions, soil retention, dissolution kinetics, and biological uptake pathways [76]. However, accurate prediction remains challenging because EWNMs exhibit highly heterogeneous compositions and dynamic transformation behaviors that differ substantially from conventional contaminants. Major uncertainties arise from limited environmental monitoring data, insufficient characterization of transformation processes, and incomplete understanding of long-term ecological interactions [77]. Future improvements in environmental fate modeling will require integration of advanced analytical techniques, field observations, machine learning approaches, and mechanistic understanding of nanoparticle behavior. Such efforts are essential for developing reliable exposure assessments and supporting evidence-based regulatory decision-making regarding EWNMs. Overall, the environmental transport and fate of EWNMs are dynamic processes controlled by both physicochemical properties and environmental conditions. Continuous transformation through aggregation, dissolution, oxidation, adsorption, and surface modification influences their persistence, mobility, exposure potential, and ecological risks. Improved understanding of these coupled transport and transformation processes is essential for developing reliable environmental monitoring strategies and risk assessment frameworks.

## 5. Physicochemical Characteristics of E-Waste-Derived Nanomaterials

Current understanding of the physicochemical characteristics of electronic waste-derived nanomaterials (EWNMs) is based on a combination of direct observations of e-waste-associated nanoparticles and broader studies of incidental and engineered nanomaterials. Although fundamental properties such as particle size, morphology, surface charge, and surface chemistry are common to all nanoscale materials, EWNMs exhibit greater compositional heterogeneity because they originate from diverse electronic components and undergo continuous environmental transformation [80]. These characteristics strongly influence their environmental transport, persistence, biological interactions, and toxicological behavior. Therefore, this section summarizes the key physicochemical properties of EWNMs while acknowledging the limited availability of source-specific experimental data.

### 5.1. Particle Size Distribution and Surface Area

Particle size is one of the most influential parameters affecting the environmental and biological behavior of EWNMs. Nanomaterials generated during electronic waste processing typically range from a few nanometers to several hundred nanometers in diameter, although larger aggregates may also be present. Mechanical fragmentation, thermal degradation, combustion, corrosion, and chemical precipitation processes generate particles with broad size distributions. As particle size decreases, specific surface area increases dramatically. This increase in surface area enhances adsorption capacity, catalytic activity, chemical reactivity, and interaction with biological membranes [81]. Smaller nanoparticles also possess greater environmental mobility and may penetrate biological barriers more efficiently than larger particles. The broad size distributions commonly observed in EWNMs contribute to variability in transport behavior, environmental persistence, and toxicity. Ultrafine particles below 100 nm are generally considered particularly important due to their increased potential for inhalation exposure and cellular uptake.

### 5.2. Surface Charge and Zeta Potential

Surface charge significantly influences nanoparticle stability, environmental transport, and biological interactions. The electrical charge present on nanoparticle surfaces determines interactions with surrounding particles, dissolved ions, natural organic matter, and cellular membranes. Zeta potential is commonly used as an indicator of nanoparticle surface charge and colloidal stability. Particles with high positive or negative zeta potential values generally remain dispersed in suspension due to electrostatic repulsion, whereas particles with low zeta potential values tend to aggregate and settle from suspension [78]. The surface charge of EWNMs is highly dynamic and may vary according to environmental conditions such as pH, ionic strength, dissolved organic matter concentration, and oxidation state. Changes in surface charge can significantly alter environmental mobility, bioavailability, and toxicological behavior.

### 5.3. Morphology and Structural Diversity

EWNMs exhibit remarkable morphological diversity due to the heterogeneous nature of electronic waste and the variety of formation mechanisms involved. Common morphologies include spherical nanoparticles, irregular fragments, rod-shaped particles, plate-like structures, fibrous materials, porous aggregates, and complex hybrid nanocomposites. Mechanical shredding typically produces irregularly shaped particles with rough surfaces, while thermal and chemical processes often generate more spherical nanoparticles through nucleation and condensation mechanisms [80]. Carbonaceous nanoparticles formed during combustion may exhibit graphitic, amorphous, or soot-like structures. Particle morphology influences sedimentation behavior, aggregation tendencies, cellular uptake efficiency, and surface reactivity. Irregular and high-aspect-ratio particles may interact differently with biological systems compared with spherical nanoparticles of similar composition.

### 5.4. Chemical Composition and Surface Functionalization

The chemical composition of EWNMs reflects the diverse material composition of electronic waste. Metallic nanoparticles derived from copper, silver, gold, nickel, chromium, cobalt, zinc, iron, manganese, and lead have been identified in recycling emissions and contaminated environments. Metal oxide nanoparticles including copper oxide, zinc oxide, iron oxide, titanium dioxide, and cobalt oxide are also frequently detected. In addition to inorganic particles, EWNMs may contain carbonaceous nanomaterials, polymer-derived nanoparticles, semiconductor fragments, and mixed nanocomposite structures [82]. Many particles retain surface-associated contaminants such as brominated flame retardants, persistent organic pollutants, plastic additives, and heavy metals. Surface functionalization occurs naturally through environmental exposure and aging processes. Adsorption of organic molecules, inorganic ions, proteins, and microbial metabolites modifies nanoparticle surface properties and influences their environmental fate and biological activity.

### 5.5. Crystallinity and Phase Structure

Crystallinity and phase structure play important roles in determining nanoparticle stability, dissolution behavior, electronic properties, and toxicity. EWNMs may exist in crystalline, amorphous, or partially crystalline forms depending on formation conditions. Thermal processes often generate highly crystalline metal oxide nanoparticles, whereas rapid cooling and mechanical fragmentation may produce amorphous or poorly ordered structures. Variations in crystal structure can significantly influence surface energy, chemical reactivity, and interactions with biological systems [83]. Different crystalline phases of the same material may exhibit distinct toxicological properties due to differences in dissolution kinetics, reactive oxygen species generation, and cellular interactions. Consequently, characterization of crystallinity is essential for accurate risk assessment of EWNMs.

### 5.6. Aggregation and Agglomeration Behavior

Aggregation and agglomeration are among the most important processes affecting the environmental fate of EWNMs. Aggregation involves the formation of strongly bound particle clusters, whereas agglomeration generally refers to weaker associations between particles. Nanoparticles rarely remain as isolated entities after environmental release. Instead, they interact with other particles, dissolved ions, organic matter, and environmental surfaces, leading to the formation of larger aggregates. Aggregation behavior is influenced by particle size, surface charge, ionic strength, pH, and natural organic matter concentrations [84]. Formation of aggregates can alter particle mobility, sedimentation rates, biological uptake, and toxicity. While aggregation may reduce nanoparticle transport in some systems, aggregate disruption can regenerate smaller particles and restore mobility under changing environmental conditions.

### 5.7. Solubility and Dissolution Kinetics

Solubility and dissolution behavior strongly influence the environmental persistence and toxicity of EWNMs. Many metal-containing nanoparticles gradually dissolve after environmental release, producing dissolved metal ions that may contribute significantly to observed biological effects. Dissolution rates depend on particle size, crystal structure, surface area, oxidation state, pH, temperature, and surrounding chemical composition. Smaller particles generally dissolve more rapidly because of their higher surface energy and greater surface area exposure [85]. The release of dissolved ions can increase toxicity through mechanisms distinct from those associated with intact nanoparticles. Consequently, both particulate and ionic forms must be considered when evaluating environmental risks associated with EWNMs.

### 5.8. Interaction with Natural Organic Matter

**Figure 6:**
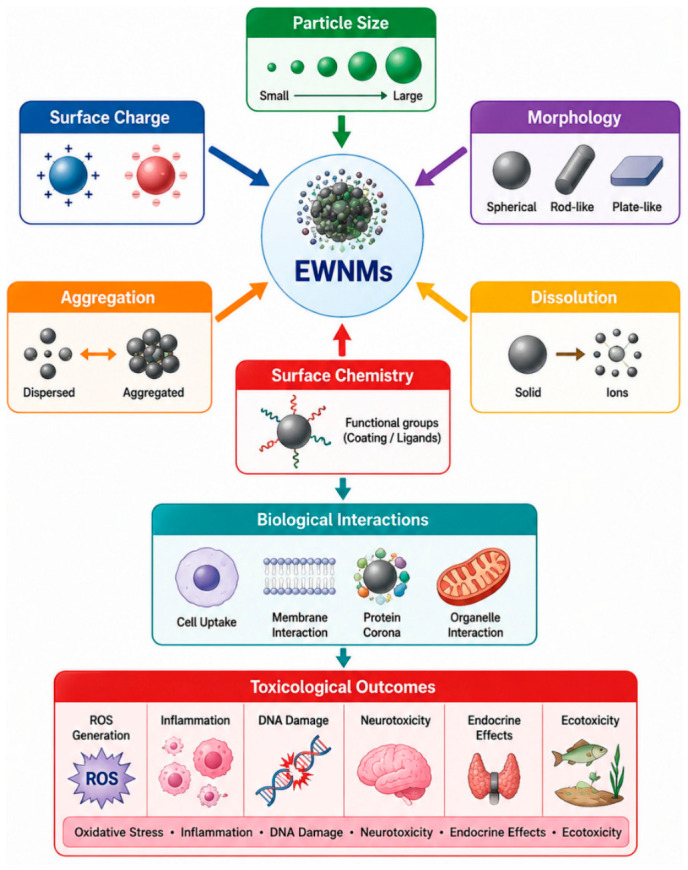
Schematic representation of the influence of particle size, surface charge, morphology, aggregation behavior, dissolution characteristics, and surface chemistry on the biological interactions and toxicological responses of electronic waste-derived nanomaterials.

Natural organic matter (NOM) plays a critical role in controlling the environmental behavior of EWNMs seen in schematic in Figure 6. Humic substances, fulvic acids, proteins, polysaccharides, and microbial exudates readily adsorb onto nanoparticle surfaces, forming environmentally derived surface coatings. These coatings can significantly alter colloidal stability, aggregation behavior, surface charge, dissolution kinetics, and biological interactions. In some cases, NOM enhances nanoparticle dispersion and transport by preventing aggregation. In other situations, bridging interactions between particles and organic matter promote aggregation and sedimentation [86]. The formation of organic coatings may also influence nanoparticle toxicity by modifying cellular uptake, reducing direct particle-cell interactions, or altering reactive oxygen species generation. Consequently, interactions between EWNMs and natural organic matter represent a critical factor governing environmental fate and ecological risk. Table 6 highlights the critical role of physicochemical properties in determining the environmental fate of EWNMs. Factors such as particle size, surface charge, morphology, crystallinity, and aggregation state directly influence dissolution kinetics and transformation processes, while environmental conditions including pH, redox potential, and ionic strength further regulate nanoparticle stability. These interactions collectively control the persistence, mobility, bioavailability, and potential ecological risks of EWNMs across terrestrial and aquatic environments.

### 5.9. Influence of Physicochemical Properties on Toxicity

The toxicity of EWNMs is strongly influenced by their physicochemical characteristics rather than solely by their chemical composition. Particle size, surface area, morphology, surface charge, crystallinity, dissolution behavior, and aggregation state collectively determine biological responses following exposure. Smaller nanoparticles generally exhibit greater cellular uptake and tissue penetration capabilities due to their nanoscale dimensions. High surface area and reactive surface sites may enhance oxidative stress generation and catalytic activity [88]. Surface charge influences interactions with cellular membranes, while dissolution processes contribute to metal ion release and associated toxicity. Morphological features such as particle shape and surface roughness may affect cellular internalization and inflammatory responses. Similarly, aggregation state influences exposure concentration and bioavailability within biological systems [87]. Environmental aging and surface modification further alter toxicological behavior by changing nanoparticle reactivity and biological interactions. The complex interplay among these physicochemical properties highlights the need for comprehensive characterization of EWNMs when assessing environmental risks and human health impacts. Future toxicological investigations should therefore integrate detailed physicochemical analyses with mechanistic biological studies to improve understanding of nanoparticle-induced adverse effects. The extent of biological uptake and the resulting toxicological responses of EWNMs depend not only on the exposure route but also on particle size, chemical composition, surface properties, exposure duration, and dose. Following inhalation, ingestion, dermal absorption, or occupational exposure, EWNMs may translocate across biological barriers, accumulate in target organs, and initiate a range of cellular and molecular responses. Consequently, understanding exposure pathways is essential for interpreting the mechanisms of toxicity and evaluating the long-term health risks associated with chronic exposure.

## 6. Toxicological Impacts on Biological Systems

Current understanding of the toxicological effects of electronic waste-derived nanomaterials (EWNMs) remains limited because relatively few studies have investigated these materials directly. Therefore, the mechanistic insights discussed in this section integrate available evidence from EWNMs, incidental nanoparticles, and engineered nanomaterials while distinguishing established findings from inferred mechanisms. Owing to their nanoscale dimensions, high surface reactivity, and ability to cross biological barriers, EWNMs can induce oxidative stress, inflammation, genotoxicity, neurotoxicity, endocrine disruption, reproductive toxicity, and ecological effects, although these responses depend on particle composition, size, surface chemistry, exposure route, and duration of exposure [17] see in Table 7.

### 6.1. Cellular Uptake Mechanisms

Cellular uptake represents the initial step governing the biological effects of EWNMs. Following exposure, nanoparticles may interact with cell membranes and enter cells through multiple pathways depending on particle characteristics and cell type [93]. Endocytosis is considered one of the primary mechanisms through which nanoparticles enter cells. Phagocytosis, macropinocytosis, clathrin-mediated endocytosis, and caveolae-mediated uptake facilitate internalization of EWNMs into intracellular compartments. Once internalized, nanoparticles may localize within endosomes, lysosomes, mitochondria, or the cytoplasm. Certain ultrasmall nanoparticles may also enter cells through passive diffusion or direct membrane translocation mechanisms. The efficiency of cellular uptake is influenced by particle size, shape, surface charge, and surface functionalization [90]. Prior to internalization, EWNMs interact directly with cellular membranes. Electrostatic interactions, hydrophobic forces, and receptor-mediated binding may alter membrane integrity and permeability. Highly reactive nanoparticles can disrupt lipid bilayers, induce membrane depolarization, and modify membrane-associated signaling pathways. Direct membrane damage may contribute to altered cellular homeostasis, increased permeability, and initiation of inflammatory responses.

### 6.2. Oxidative Stress and Reactive Oxygen Species Generation

Oxidative stress is widely recognized as one of the central mechanisms underlying nanoparticle toxicity. Numerous EWNMs possess redox-active surfaces capable of generating reactive oxygen species (ROS) either directly or indirectly through cellular interactions. Mitochondria are particularly vulnerable to nanoparticle-induced oxidative damage [95]. Internalized EWNMs may accumulate within mitochondria and interfere with electron transport chain function, resulting in excessive ROS production and impaired energy metabolism. Mitochondrial dysfunction can lead to reduced ATP synthesis, altered membrane potential, calcium imbalance, and activation of apoptotic signaling pathways. Persistent mitochondrial damage has been associated with chronic cellular stress and tissue injury. Reactive oxygen species generated by EWNMs can attack membrane lipids, initiating lipid peroxidation reactions [91]. Oxidative degradation of membrane phospholipids compromises membrane integrity and produces reactive aldehydes that further amplify cellular damage. Lipid peroxidation contributes to membrane dysfunction, altered cellular signaling, and increased susceptibility to apoptosis and necrosis. Proteins are major cellular targets of oxidative stress induced by EWNMs [96]. ROS-mediated oxidation may alter protein structure, enzymatic activity, receptor function, and intracellular signaling processes. Protein oxidation can impair cellular metabolism, disrupt antioxidant defense systems, and contribute to accumulation of damaged proteins associated with various pathological conditions.

### 6.3. Inflammation and Immune Responses

Inflammatory responses represent another major consequence of nanoparticle exposure. Activation of immune cells and inflammatory signaling pathways can occur following recognition of EWNMs as foreign or harmful agents. Exposure to EWNMs stimulates production of pro-inflammatory cytokines including tumor necrosis factor-alpha (TNF-α), interleukin-1β (IL-1β), interleukin-6 (IL-6), and various chemokines [97]. These mediators regulate immune responses and recruit inflammatory cells to sites of nanoparticle accumulation. Excessive cytokine production may contribute to tissue injury, immune dysregulation, and chronic inflammatory conditions. Several studies have demonstrated activation of nuclear factor kappa B (NF-κB) and mitogen-activated protein kinase (MAPK) pathways following nanoparticle exposure [89]. These signaling cascades regulate expression of inflammatory genes, cytokines, adhesion molecules, and stress-response proteins. Persistent activation of these pathways may promote chronic inflammation, oxidative stress, and cellular dysfunction. Long-term exposure to EWNMs may result in sustained inflammatory responses that contribute to chronic disease development [92]. Persistent inflammation has been associated with fibrosis, pulmonary injury, cardiovascular dysfunction, immune disturbances, and cancer progression. The risk of chronic inflammation is particularly relevant in occupational settings where repeated exposure to airborne nanoparticles may occur over extended periods.

### 6.4. Genotoxicity and DNA Damage

Genotoxic effects are among the most concerning biological consequences associated with EWNM exposure because they may contribute to carcinogenesis and heritable genetic alterations. Nanoparticle-induced ROS generation can directly damage DNA molecules, resulting in single-strand and double-strand breaks [94]. Such damage interferes with DNA replication and transcription processes and may trigger apoptosis if repair mechanisms fail. Numerous metal-containing nanoparticles derived from electronic waste have demonstrated the ability to induce oxidative DNA damage in both in vitro and in vivo systems. Exposure to EWNMs has been associated with chromosomal instability, micronucleus formation, chromosomal fragmentation, and mitotic abnormalities [98]. These alterations may arise from direct nanoparticle interactions with genetic material or secondary effects associated with oxidative stress and inflammation [99]. Beyond direct genetic damage, EWNMs may induce epigenetic alterations that influence gene expression without changing DNA sequence. Changes in DNA methylation, histone modification, and non-coding RNA regulation have been reported following nanoparticle exposure. Such modifications may contribute to long-term biological effects and influence susceptibility to disease development.

### 6.5. Neurotoxicity

Neurotoxicity has emerged as a significant concern because many nanoparticles possess the ability to reach the central nervous system and interact with neural tissues [100]. Due to their small size, certain EWNMs may cross the blood–brain barrier through transcellular transport, paracellular pathways, or transport along olfactory neurons. Once inside the brain, nanoparticles may accumulate within neural tissues and interact with neurons, astrocytes, and microglia. Accumulation of nanoparticles in the nervous system may activate microglial cells and induce neuroinflammatory responses. Elevated production of inflammatory mediators and oxidative stress can disrupt neuronal function and promote neural tissue damage. Chronic neuroinflammation is increasingly recognized as an important mechanism contributing to neurological disorders [101]. Experimental evidence suggests that nanoparticle exposure may contribute to pathological processes associated with neurodegenerative diseases. Oxidative stress, mitochondrial dysfunction, protein aggregation, and chronic inflammation may collectively promote neuronal injury and cognitive impairment. Although epidemiological evidence remains limited, concerns regarding long-term neurological consequences warrant further investigation.

### 6.6. Endocrine Disruption

Electronic waste contains numerous substances capable of interfering with endocrine regulation, including heavy metals, brominated flame retardants, plastic additives, and nanoparticle-associated contaminants. EWNMs may influence hormone synthesis, secretion, transport, receptor binding, and metabolic pathways. Experimental studies have reported alterations in thyroid hormone regulation, steroidogenesis, reproductive hormone levels, and metabolic signaling following exposure to certain metal-containing nanoparticles [102]. Endocrine disruption may contribute to developmental abnormalities, reproductive dysfunction, metabolic disorders, and immune disturbances.

### 6.7. Reproductive and Developmental Toxicity

**Figure 7:**
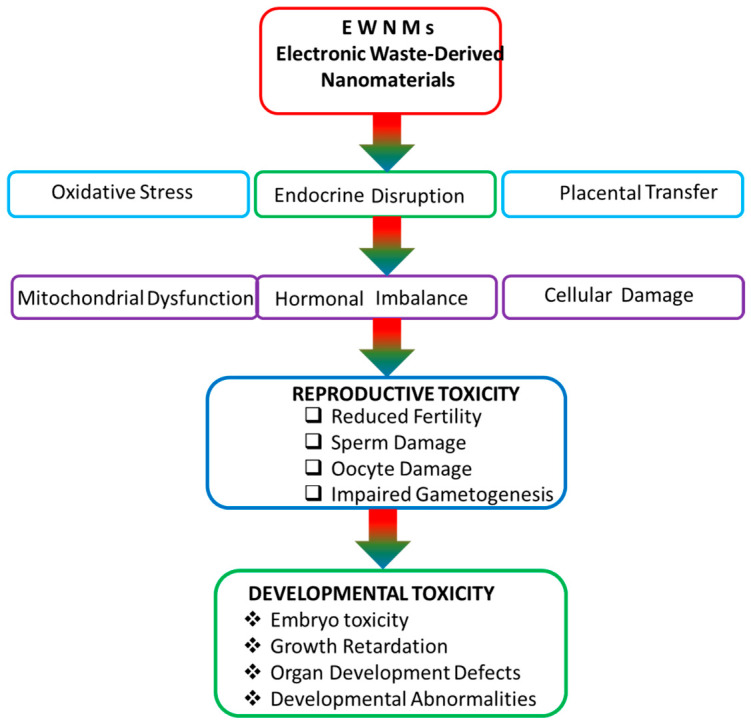
Schematic illustration of the biological mechanisms through which EWNM exposure induces oxidative stress, endocrine disruption, mitochondrial dysfunction, and placental transfer, ultimately contributing to reproductive impairment, embryo toxicity, and developmental abnormalities.

Reproductive systems are particularly sensitive to nanoparticle-induced toxicity because of the importance of cellular integrity, hormonal regulation, and developmental processes. The workflow is summarized in Figure 7 for better understanding. Many studies have demonstrated that exposure to metal-containing nanoparticles may adversely affect spermatogenesis, sperm quality, testosterone production, and testicular structure. Oxidative stress and inflammatory responses are considered major mechanisms contributing to male reproductive toxicity. In females, EWNMs may interfere with ovarian function, follicular development, hormone regulation, and reproductive cycles [103]. Nanoparticle accumulation within reproductive tissues may alter cellular signaling and impair fertility-related processes. Certain nanoparticles possess the ability to cross placental barriers and reach developing embryos. Prenatal exposure has been associated with developmental abnormalities, growth impairment, oxidative stress, and altered organ development in experimental models. The developing fetus is considered particularly vulnerable because of rapid cellular proliferation and limited detoxification capacity [104].

### 6.8. Ecotoxicological Effects

In addition to human health concerns, EWNMs may significantly affect ecological systems through interactions with microorganisms, plants, and aquatic organisms. Aquatic organisms are highly susceptible to nanoparticle exposure because water serves as a major transport medium for EWNMs [105]. Fish, algae, crustaceans, mollusks, and aquatic microorganisms may experience oxidative stress, developmental abnormalities, behavioral changes, impaired reproduction, and mortality following exposure. Bioaccumulation within aquatic food webs raises concerns regarding trophic transfer and ecosystem-level impacts [106]. Soil microorganisms play critical roles in nutrient cycling, organic matter decomposition, and ecosystem functioning. Exposure to EWNMs may alter microbial diversity, enzymatic activity, and community structure. Changes in microbial populations may influence soil fertility, nutrient availability, and ecological stability within contaminated environments. Plants may absorb EWNMs through root systems and accumulate nanoparticles within tissues [107]. Exposure has been associated with reduced seed germination, impaired photosynthesis, oxidative stress, altered nutrient uptake, and growth inhibition. Plant accumulation of EWNMs may additionally facilitate transfer of nanoscale contaminants into terrestrial food chains. Table 8 summarizes the major genotoxic effects associated with EWNM exposure. Current evidence indicates that oxidative stress plays a central role in EWNM-induced genotoxicity by promoting DNA strand breaks, oxidative base modifications, and chromosomal damage. In addition to direct genetic injury, EWNMs may alter epigenetic regulation, microRNA expression, and DNA repair processes, thereby amplifying long-term cellular dysfunction. These mechanisms contribute to genomic instability and may increase the risk of chronic diseases, reproductive disorders, and carcinogenic outcomes following prolonged exposure to nanoscale contaminants derived from electronic waste.

### 6.9. Toxicological Challenges and Knowledge Gaps

Despite growing evidence regarding the biological effects of EWNMs, significant knowledge gaps remain. Most toxicological studies have focused on individual nanoparticles under controlled laboratory conditions, whereas environmental exposures typically involve complex mixtures of particles, metals, organic pollutants, and transformation products. Long-term chronic exposure data remain limited, particularly for environmentally aged nanoparticles and realistic exposure scenarios [108]. Furthermore, variability in particle composition, surface chemistry, and environmental transformation complicates comparison among studies and hinders development of standardized risk assessment frameworks. Additional research is needed to clarify dose–response relationships, mechanisms of chronic toxicity, trophic transfer processes, vulnerable populations, and combined effects of multiple contaminants. Integration of advanced toxicological models, omics technologies, and environmentally relevant exposure systems will be essential for improving understanding of the health and ecological risks associated with electronic waste-derived nanomaterials. Although acute exposure studies provide valuable mechanistic insights, increasing attention is being directed toward the potential consequences of long-term, low-dose exposure to EWNMs. Chronic exposure may result in persistent oxidative stress, prolonged inflammatory responses, cumulative cellular damage, altered immune function, and progressive bioaccumulation in specific organs, potentially increasing the risk of chronic respiratory, neurological, cardiovascular, and reproductive disorders. However, long-term in vivo studies and epidemiological evidence remain limited, highlighting the need for extended exposure assessments to improve environmental and human health risk evaluation.

## 7. Human Exposure Pathways and Public Health Risks

Human exposure to electronic waste-derived nanomaterials (EWNMs) has emerged as a growing public health concern due to the rapid expansion of electronic waste generation and recycling activities worldwide [35]. Unlike conventional pollutants, nanoscale contaminants possess unique physicochemical characteristics that facilitate environmental mobility, biological uptake, and penetration of physiological barriers. Consequently, exposure may occur through multiple routes including inhalation, ingestion, dermal contact, and occupational activities. While recycling workers represent the most highly exposed population, nearby residents and the general public may also experience chronic exposure through contaminated environmental media and food chains [109]. The potential health consequences of EWNM exposure are particularly significant because nanoparticles can accumulate in tissues, induce oxidative stress, trigger inflammation, and interact with critical biological systems even at relatively low concentrations. Beyond direct toxicity to individual organisms, EWNMs may undergo trophic transfer through terrestrial and aquatic food webs following uptake by microorganisms, plants, and invertebrates. Such transfer may enhance bioaccumulation across higher trophic levels and alter ecosystem structure and function. Although current evidence remains limited, the potential ecological consequences of long-term environmental exposure warrant further investigation to support comprehensive ecological risk assessment.

### 7.1. Occupational Exposure in Recycling Facilities

Occupational exposure represents one of the most significant sources of human contact with EWNMs. Workers involved in dismantling, shredding, crushing, sorting, metal recovery, thermal treatment, and chemical extraction activities are routinely exposed to airborne nanoparticles generated during recycling operations [110]. Consequently, workers may experience prolonged exposure to complex mixtures of metallic nanoparticles, metal oxides, polymer fragments, carbonaceous nanomaterials, and associated toxic contaminants. Exposure levels are generally highest during mechanical processing and open burning operations where large quantities of ultrafine particles are released into the surrounding environment [111]. The magnitude of risk depends on exposure duration, particle characteristics, workplace conditions, and individual susceptibility.

### 7.2. Inhalation Exposure

Inhalation is widely regarded as the primary route of human exposure to EWNMs because airborne nanoparticles are readily generated during recycling and waste management activities. Their small size enables prolonged atmospheric suspension and deep penetration into the respiratory system. The deposition of inhaled nanoparticles within the respiratory tract is strongly influenced by particle size, shape, density, and surface characteristics [112]. Larger particles tend to deposit in the upper airways, whereas ultrafine nanoparticles can penetrate deeply into the bronchioles and alveolar regions of the lungs. Once deposited, nanoparticles may interact with epithelial cells, alveolar macrophages, and pulmonary tissues. Some particles may subsequently translocate across the air-blood barrier and enter systemic circulation, facilitating distribution to secondary organs including the liver, kidneys, heart, and brain [113]. The high deposition efficiency of ultrafine particles increases the potential for long-term biological effects. Pulmonary tissues are among the first biological targets following inhalation exposure. Experimental studies indicate that inhaled nanoparticles can induce oxidative stress, inflammation, epithelial injury, and impaired pulmonary function. Metal-containing EWNMs may generate reactive oxygen species and activate inflammatory signaling pathways, leading to cytokine production and recruitment of immune cells. Chronic exposure may contribute to airway remodeling, fibrosis, reduced lung function, and increased susceptibility to respiratory diseases [114]. Individuals with pre-existing respiratory conditions may be particularly vulnerable to these effects.

### 7.3. Dermal Exposure and Skin Penetration

Dermal exposure occurs through direct contact with contaminated electronic waste, recycling residues, dust particles, wastewater, and processing chemicals. Recycling workers frequently handle electronic components without adequate protective equipment, increasing opportunities for skin exposure. The ability of nanoparticles to penetrate the skin depends on particle size, surface chemistry, skin condition, and duration of exposure [115]. Although intact skin provides an effective barrier against many contaminants, damaged or compromised skin may permit greater nanoparticle penetration. Hair follicles, sweat glands, and microabrasions may also serve as potential entry pathways. In addition to direct penetration, dermal exposure may result in localized oxidative stress, inflammation, irritation, and allergic responses. Nanoparticles deposited on the skin can also contribute indirectly to exposure through hand-to-mouth transfer and accidental ingestion.

### 7.4. Oral Exposure and Dietary Intake

Oral exposure represents another important pathway through which humans may encounter EWNMs. Contamination of drinking water, agricultural products, aquatic organisms, and food-processing environments can facilitate nanoparticle ingestion. Electronic waste recycling facilities and disposal sites may release nanoparticles into surface waters, groundwater systems, and drinking water sources [116]. Wastewater discharges, landfill leachates, and atmospheric deposition contribute to contamination of aquatic environments. Consumption of contaminated water may introduce EWNMs into the gastrointestinal tract, where nanoparticles interact with intestinal epithelial cells, microbiota, and digestive fluids. Certain particles may cross intestinal barriers and enter systemic circulation, enabling distribution throughout the body [117]. Accumulation of EWNMs within plants, aquatic organisms, and livestock creates opportunities for food-chain exposure [118]. Nanoparticles absorbed by crops may accumulate in edible tissues, while aquatic organisms may bioaccumulate contaminants through waterborne exposure. Consumption of contaminated fish, shellfish, vegetables, grains, and animal products may therefore contribute to long-term dietary exposure. Although the extent of trophic transfer remains under investigation, available evidence suggests that food chains may represent an important route of chronic human exposure in contaminated regions.

### 7.5. Exposure Among Vulnerable Populations

Certain population groups may experience greater susceptibility to EWNM exposure due to physiological, developmental, or health-related factors. Children are considered among the most vulnerable populations because of their developing organ systems, higher metabolic rates, and greater intake of air, food, and water relative to body weight [119]. Hand-to-mouth behaviors and frequent contact with contaminated surfaces may further increase exposure. Nanoparticle exposure during childhood may interfere with neurological development, immune function, respiratory health, and endocrine regulation. Early-life exposure may also increase susceptibility to chronic diseases later in life. Pregnant women represent another high-risk group because nanoparticles may affect both maternal health and fetal development. Certain nanoparticles have demonstrated the ability to cross placental barriers and reach developing embryos. Prenatal exposure may contribute to oxidative stress, inflammatory responses, altered fetal growth, developmental abnormalities, and disruption of normal developmental processes. Potential effects on pregnancy outcomes remain an important area of ongoing investigation. Elderly individuals often exhibit reduced physiological resilience and increased prevalence of chronic diseases, making them more susceptible to environmental contaminants [120]. Age-related declines in pulmonary function, immune competence, and cardiovascular health may increase sensitivity to nanoparticle-induced toxicity. Chronic exposure to EWNMs may exacerbate existing medical conditions and contribute to age-related disease progression.

### 7.6. Chronic Disease Associations

**Figure 8:**
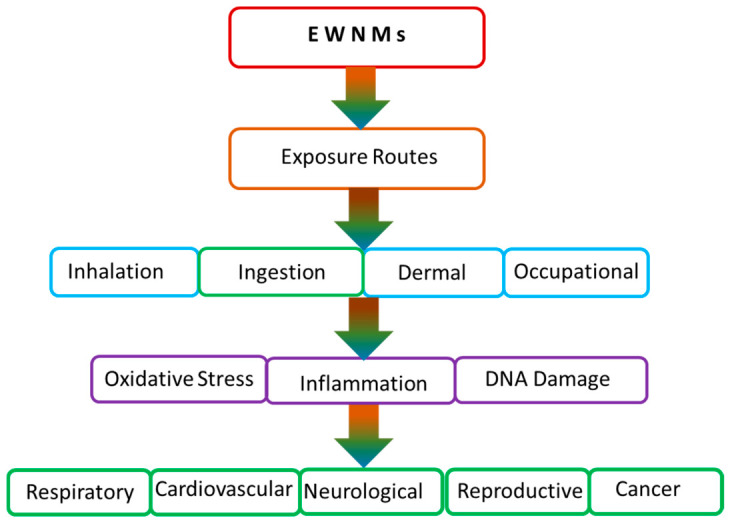
Schematic representation of environmental exposure pathways, biological responses, and major chronic health outcomes associated with prolonged EWNM exposure, including respiratory, cardiovascular, neurological, reproductive, and carcinogenic effects.

Growing evidence suggests that prolonged exposure to nanoscale pollutants may contribute to the development or progression of various chronic diseases. Respiratory effects are among the most frequently reported health outcomes associated with nanoparticle exposure. Chronic inhalation may contribute to asthma, chronic bronchitis, chronic obstructive pulmonary disease (COPD), pulmonary fibrosis, and reduced lung function. Persistent oxidative stress and inflammation within respiratory tissues are considered major mechanisms driving disease development [121] as seen in Figure 8. Nanoparticles that enter systemic circulation may interact with vascular tissues, blood cells, and endothelial surfaces. These interactions can promote oxidative stress, endothelial dysfunction, inflammation, altered coagulation, and vascular injury [122]. The ability of certain nanoparticles to reach the central nervous system has raised concerns regarding neurological health. Chronic exposure may contribute to neuroinflammation, oxidative stress, mitochondrial dysfunction, and neuronal damage. Although epidemiological evidence remains limited, nanoparticle exposure has been associated with cognitive impairment and mechanisms implicated in neurodegenerative disorders [123]. Certain metal-containing nanoparticles possess the ability to damage DNA and interfere with cellular repair mechanisms. Long-term occupational exposure to nanoparticle-rich environments may therefore increase cancer risk, although definitive epidemiological evidence remains insufficient and requires further investigation [124].

### 7.7. Epidemiological Evidence and Public Health Challenges

Epidemiological studies conducted in electronic waste recycling regions have reported elevated levels of heavy metals, inflammatory biomarkers, oxidative stress indicators, endocrine disturbances, and adverse health outcomes among exposed populations. However, distinguishing the specific contribution of EWNMs from other coexisting contaminants remains challenging because electronic waste environments contain complex mixtures of pollutants [125]. Most available investigations focus on bulk contaminants rather than nanoscale fractions, resulting in substantial uncertainty regarding exposure-response relationships. From a public health perspective, major challenges include inadequate occupational protection, limited environmental monitoring, insufficient regulatory frameworks, and lack of awareness regarding nanoparticle-related risks [126]. Improved surveillance systems, biomonitoring programs, exposure assessment methodologies, and longitudinal health studies are needed to better understand the public health implications of EWNMs. Future research should integrate environmental measurements, toxicological data, and epidemiological evidence to support development of evidence-based risk management strategies and protective regulatory policies.

Table 9 highlights several critical challenges that currently limit comprehensive evaluation of EWNM-related public health risks. Significant uncertainties remain regarding exposure quantification, long-term health outcomes, biomarker development, and the susceptibility of vulnerable populations. The lack of standardized monitoring approaches and EWNM-specific exposure guidelines further complicates risk assessment and occupational protection efforts. Addressing these gaps through multidisciplinary epidemiological, toxicological, and biomonitoring studies will be essential for developing evidence-based regulatory frameworks and effective public health interventions.

## 8. Advanced Detection and Characterization Techniques

Accurate detection and characterization of electronic waste-derived nanomaterials (EWNMs) are fundamental for evaluating their environmental occurrence, transport, exposure pathways, and potential health risks. Current analytical approaches are largely adapted from methodologies originally developed for engineered nanomaterials and ultrafine particles; however, their application to EWNMs is complicated by the heterogeneous composition, diverse physicochemical characteristics, and continuous environmental transformations of these materials [127]. Reliable identification and monitoring of EWNMs therefore require the complementary use of microscopic, spectroscopic, elemental, and physicochemical techniques, as no single analytical method can comprehensively characterize their properties across complex environmental matrices. The major analytical techniques used for the detection and characterization of EWNMs, together with their capabilities, limitations, and environmental applicability, are summarized in Table 10.

### 8.1. Challenges in EWNM Detection and Characterization

Detection of EWNMs presents several technical and methodological challenges. First, environmental concentrations are often low, and nanoparticles are distributed within highly complex matrices such as air, soil, sediments, wastewater, and biological tissues. Second, EWNMs undergo continuous transformation through aggregation, dissolution, oxidation, sulfidation, and surface modification, making their characterization highly dynamic. Another major challenge is distinguishing EWNMs from naturally occurring nanoparticles and engineered nanomaterials present in the environment [128]. Sample preparation procedures may also alter particle properties and introduce analytical artifacts. Furthermore, the absence of standardized analytical protocols limits comparability among studies and complicates environmental monitoring efforts. Consequently, comprehensive characterization generally requires the application of multiple complementary techniques capable of providing information on morphology, composition, size distribution, crystallinity, surface chemistry, and elemental content.

### 8.2. Electron Microscopy Techniques

Electron microscopy remains one of the most important analytical approaches for direct visualization of EWNMs. These techniques provide detailed information regarding particle morphology, size, aggregation state, and structural characteristics. Transmission electron microscopy (TEM) is widely regarded as one of the most powerful tools for nanoparticle characterization. TEM provides high-resolution imaging at nanometer and sub-nanometer scales, enabling direct observation of particle size, shape, crystallinity, and internal structure. For EWNMs, TEM is frequently used to characterize metallic nanoparticles, metal oxides, carbonaceous nanomaterials, and mixed nanocomposites. Coupling TEM with selected area electron diffraction (SAED) and energy-dispersive X-ray spectroscopy (EDS) further enables structural and elemental analysis of individual particles [129]. The major advantages of TEM include exceptional spatial resolution and detailed structural information. However, sample preparation can be time-consuming, and the technique typically analyzes only a limited number of particles, which may affect representativeness. Scanning electron microscopy (SEM) is extensively used for examining nanoparticle morphology, surface texture, and particle aggregation behavior. SEM generates high-resolution images by scanning a focused electron beam across sample surfaces. Compared with TEM, SEM generally requires less complex sample preparation and is particularly useful for analyzing heterogeneous environmental samples containing large particle populations [130]. Coupling SEM with EDS enables qualitative and quantitative elemental characterization of EWNMs. SEM has been widely applied to investigate nanoparticles generated during shredding, combustion, thermal treatment, and environmental weathering of electronic waste materials.

### 8.3. Spectroscopic Techniques

Spectroscopic methods provide valuable information regarding chemical composition, molecular structure, surface chemistry, and functional groups associated with EWNMs. Raman spectroscopy is a non-destructive analytical technique widely used for characterizing carbonaceous nanomaterials, polymer fragments, and semiconductor particles. The technique measures molecular vibrational modes, enabling identification of chemical structures and material composition. In EWNM studies, Raman spectroscopy is particularly useful for detecting graphitic carbon, soot particles, carbon nanotube-like structures, and polymer degradation products generated during thermal treatment and combustion processes. The method offers rapid analysis with minimal sample preparation and can be combined with microscopy for spatially resolved characterization of individual particles. Fourier transform infrared spectroscopy (FTIR) is commonly employed to identify functional groups and molecular structures present on nanoparticle surfaces. FTIR provides information regarding organic coatings, polymer composition, oxidation products, and surface modifications resulting from environmental aging. The technique is especially useful for characterizing plastic-derived nanoparticles and investigating interactions between EWNMs and natural organic matter [131]. FTIR analysis can also reveal chemical changes occurring during environmental weathering and degradation processes. X-ray photoelectron spectroscopy (XPS) is a surface-sensitive technique used to determine elemental composition, oxidation state, and chemical bonding environments. Because nanoparticle behavior is strongly influenced by surface properties, XPS plays a critical role in EWNM characterization. XPS can identify oxidation states of metals, quantify surface contaminants, and evaluate chemical transformations occurring during environmental exposure. The technique is particularly valuable for investigating corrosion products and environmentally aged nanoparticles.

### 8.4. Particle Size and Surface Analysis Methods

Particle size distribution and colloidal behavior are key determinants of nanoparticle transport, bioavailability, and toxicity. Several analytical techniques have been developed to measure these properties. Dynamic light scattering (DLS) is one of the most widely used techniques for determining nanoparticle size distribution in liquid suspensions. The method measures fluctuations in scattered light resulting from Brownian motion of suspended particles. DLS provides rapid analysis and is particularly useful for evaluating aggregation behavior, hydrodynamic diameter, and colloidal stability of EWNMs. However, the technique may be less accurate for highly polydisperse or irregularly shaped particle populations commonly encountered in environmental samples [132]. Nanoparticle tracking analysis (NTA) enables visualization and tracking of individual particles suspended in liquids. By analyzing particle movement under Brownian motion, NTA provides size distribution and particle concentration measurements. Compared with DLS, NTA often offers improved resolution for heterogeneous nanoparticle populations. The technique has become increasingly valuable for characterizing environmental colloids and complex EWNM suspensions.

### 8.5. Elemental and Chemical Quantification

Quantitative determination of elemental composition is essential for identifying contamination sources and evaluating exposure risks associated with EWNMs. Inductively coupled plasma mass spectrometry (ICP-MS) is among the most sensitive analytical techniques available for trace elemental analysis. The method enables detection of metals and metalloids at extremely low concentrations. Single-particle ICP-MS (spICP-MS) represents an important advancement that allows detection and characterization of individual nanoparticles. In addition to elemental concentration, spICP-MS can provide information regarding particle size distribution and particle number concentration [133]. These techniques are widely used for monitoring metal-containing EWNMs in environmental samples, biological tissues, and occupational exposure studies. Atomic absorption spectroscopy (AAS) remains a valuable tool for quantifying metal concentrations in environmental and biological samples. Although AAS does not directly characterize nanoparticles, it provides important information regarding total metal content and dissolution products associated with EWNMs. The technique is frequently used alongside microscopy and spectroscopic methods to evaluate environmental contamination and exposure levels.

### 8.6. Environmental Monitoring Approaches

Effective environmental monitoring is necessary for assessing environmental release, transport, and exposure risks associated with EWNMs. Airborne nanoparticles generated during recycling and waste management activities are commonly monitored using filtration systems, cascade impactors, diffusion samplers, condensation particle counters, and scanning mobility particle sizers [134]. These instruments provide information regarding particle concentration, size distribution, temporal variation, and occupational exposure levels. Combined with microscopic and chemical analyses, air sampling data contribute to comprehensive exposure assessment programs. Monitoring of EWNMs in aquatic and terrestrial environments requires specialized sampling and extraction protocols [135]. Water samples are typically analyzed for suspended nanoparticles, dissolved metal species, and colloidal fractions. Soil and sediment investigations often involve sequential extraction procedures, microscopic characterization, and elemental analysis. Sampling strategies must account for spatial heterogeneity, environmental transformation processes, and temporal variations in contamination levels. Recent advances in biosensor technology have enabled development of highly sensitive platforms for nanoparticle detection [136]. Biosensors utilize biological recognition elements such as enzymes, antibodies, nucleic acids, or microbial systems to detect specific contaminants. Electrochemical, optical, fluorescence-based, and nanomaterial-enhanced biosensors offer rapid and potentially field-deployable monitoring solutions. Such technologies may facilitate real-time assessment of EWNM contamination in environmental and occupational settings.

### 8.7. Emerging AI-Assisted Analytical Technologies

**Figure 9:**
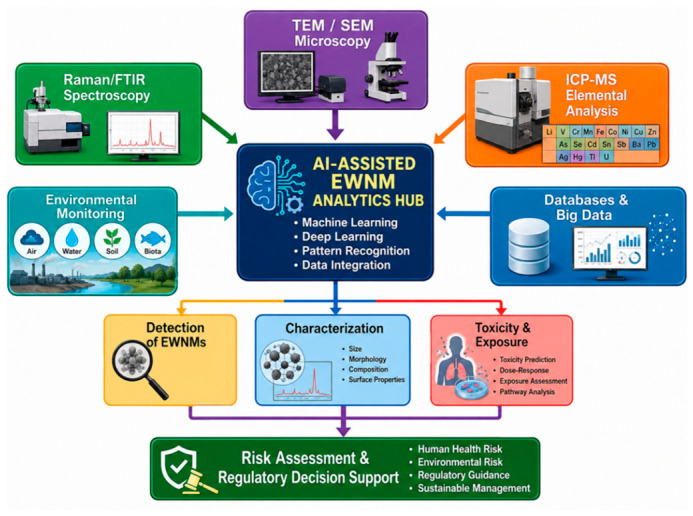
Schematic overview illustrating the integration of advanced analytical techniques, environmental monitoring platforms, artificial intelligence algorithms, and predictive modeling tools for the detection, characterization, exposure assessment, and risk evaluation of electronic waste-derived nanomaterials.

Artificial intelligence (AI) and machine learning technologies are increasingly transforming nanoparticle characterization and environmental monitoring. These approaches enable rapid analysis of large datasets generated through microscopy, spectroscopy, environmental monitoring, and toxicological investigations see in Figure 9. Machine learning algorithms can assist in automated particle recognition, image segmentation, morphology classification, spectral interpretation, and source identification [137]. AI-assisted image analysis significantly reduces processing time for TEM and SEM datasets while improving consistency and reproducibility. Advanced predictive models are also being developed to estimate nanoparticle transport, environmental fate, transformation behavior, and toxicity based on physicochemical characteristics [138]. Integration of AI with high-throughput analytical platforms may facilitate real-time environmental monitoring and risk assessment. Despite their promise, AI-assisted technologies face challenges related to data quality, model validation, standardization, and interpretability. Future research should focus on developing robust databases, harmonized analytical protocols, and explainable machine learning frameworks capable of supporting regulatory decision-making and environmental management of EWNMs. No individual analytical technique can fully characterize the physicochemical complexity of EWNMs in environmental samples. Integrating microscopic, spectroscopic, elemental, and surface analytical methods provides more comprehensive information on particle composition, morphology, chemical transformations, and environmental behavior, thereby improving exposure assessment and environmental monitoring.

## 9. Regulatory Challenges and Risk Assessment

The rapid growth of electronic waste generation and the increasing recognition of electronic waste-derived nanomaterials (EWNMs) as emerging environmental contaminants have created significant regulatory and risk assessment challenges worldwide. Existing environmental regulations were primarily developed to address bulk pollutants, hazardous chemicals, and conventional waste streams, with limited consideration of nanoscale materials generated during recycling, disposal, and environmental degradation processes. Consequently, current regulatory frameworks often fail to adequately address the unique physicochemical properties, environmental behavior, and toxicological characteristics of EWNMs [128]. Risk assessment of EWNMs is further complicated by their heterogeneous composition, dynamic environmental transformations, diverse exposure pathways, and limited availability of long-term toxicological data. As a result, substantial uncertainties remain regarding environmental exposure limits, occupational safety requirements, monitoring strategies, and regulatory management approaches. Addressing these challenges requires coordinated international efforts aimed at integrating nanotechnology, environmental science, public health, and waste management perspectives into future regulatory frameworks. Current international regulations provide an important foundation for electronic waste management and nanomaterial safety, although their primary focus remains on conventional hazardous substances and intentionally manufactured nanomaterials. The European Union has established comprehensive regulatory frameworks through the Waste Electrical and Electronic Equipment (WEEE) and Restriction of Hazardous Substances (RoHS) Directives, which promote environmentally sound recycling, hazardous substance control, and extended producer responsibility. In the United States, the USEPA applies risk-based approaches for hazardous waste management while supporting the evaluation of emerging contaminants. The OECD has developed internationally recognized guidance for nanomaterial characterization, environmental fate assessment, and safety testing that can inform future evaluation of EWNMs. At the international level, the Basel Convention regulates the transboundary movement of hazardous electronic waste and promotes environmentally sound waste management practices. Although these frameworks contribute substantially to electronic waste governance and nanomaterial risk assessment, none specifically addresses the unique characteristics, environmental behavior, exposure pathways, and risk assessment requirements of electronic waste-derived nanomaterials.

### 9.1. Current International Regulatory Frameworks

Several international agreements and regulatory programs govern electronic waste management and hazardous substances associated with electronic products. Although these frameworks play important roles in reducing environmental contamination, most were developed before the emergence of concerns regarding EWNMs. The Basel Convention is the principal international agreement regulating the transboundary movement and disposal of hazardous waste [139]. Adopted in 1989, the convention aims to minimize hazardous waste generation, promote environmentally sound waste management, and prevent the transfer of hazardous wastes from developed to developing countries. Electronic waste has increasingly been incorporated into Basel Convention discussions because of its growing environmental significance. The convention establishes controls on international shipments of e-waste and encourages responsible recycling practices. However, the framework primarily focuses on bulk hazardous materials and does not specifically address nanoscale contaminants generated during electronic waste processing. The Waste Electrical and Electronic Equipment Directive (WEEE Directive) represents one of the most comprehensive regulatory frameworks for electronic waste management [140]. The directive promotes collection, recycling, recovery, and environmentally sound disposal of waste electrical and electronic equipment. The WEEE Directive establishes producer responsibility requirements and recycling targets aimed at reducing environmental impacts associated with electronic waste. By improving collection and recycling efficiency, the directive indirectly contributes to reducing uncontrolled release of EWNMs into the environment. Nevertheless, specific monitoring and regulation of nanoscale pollutants remain largely absent from current implementation frameworks. The Restriction of Hazardous Substances Directive restricts the use of certain hazardous substances in electrical and electronic equipment. Regulated substances include lead, mercury, cadmium, hexavalent chromium, polybrominated biphenyls, and polybrominated diphenyl ethers. RoHS regulations have significantly reduced the presence of several hazardous chemicals in modern electronic products, thereby decreasing potential environmental contamination during disposal and recycling. However, these regulations primarily focus on bulk chemical constituents rather than nanoparticles generated during material degradation and waste processing activities. Collectively, these international frameworks provide complementary approaches for electronic waste management and nanomaterial governance. The European Union emphasizes product stewardship, hazardous substance restriction, and recycling efficiency through legally binding directives, whereas the United States Environmental Protection Agency (USEPA) adopts a risk-based approach that integrates hazardous waste management, pollution prevention, and environmental protection. The Organisation for Economic Co-operation and Development (OECD) supports international harmonization by developing guidance for nanomaterial characterization, environmental fate evaluation, and safety testing, while the Basel Convention focuses primarily on controlling the transboundary movement of hazardous electronic waste and promoting environmentally sound management practices. Although these frameworks have substantially improved electronic waste governance and chemical safety, they were not specifically developed to address the unique characteristics of electronic waste-derived nanomaterials (EWNMs). Standardized protocols for EWNM identification, environmental monitoring, exposure assessment, and risk evaluation therefore remain limited, highlighting the need for greater international coordination and the incorporation of source-specific nanoscale contaminants into future regulatory policies.

### 9.2. Limitations in Existing Nanomaterial Regulations

Although regulatory attention toward engineered nanomaterials has increased over the past decade, existing nanomaterial regulations remain insufficient for addressing EWNMs. Most current frameworks focus on intentionally manufactured nanomaterials used in commercial products rather than incidentally generated nanoparticles originating from waste streams. One major limitation is the absence of regulatory definitions specifically addressing EWNMs. Existing legislation often classifies contaminants according to chemical composition while overlooking nanoscale properties that may significantly influence toxicity and environmental behavior [141]. Furthermore, regulatory thresholds are generally based on mass concentrations, which may not adequately reflect nanoparticle exposure risks. Parameters such as particle number concentration, surface area, morphology, and surface reactivity are rarely incorporated into current regulatory approaches despite their importance in determining biological effects. Another challenge involves the lack of harmonized international standards for nanoparticle characterization, environmental monitoring, and exposure assessment. These gaps hinder effective implementation of nanoparticle-specific regulations and complicate comparison among regulatory programs.

### 9.3. Challenges in EWNM Risk Assessment

Comprehensive risk assessment of EWNMs remains a significant scientific and regulatory challenge. Traditional risk assessment frameworks were designed for conventional chemicals and bulk particulate contaminants and may not adequately capture the complexity of nanoscale pollutants. One of the most significant obstacles in EWNM risk assessment is the absence of standardized testing methodologies. Variations in sample preparation, characterization techniques, exposure conditions, toxicity endpoints, and reporting practices often produce inconsistent results across studies. Differences in particle size, aggregation state, surface chemistry, and environmental transformations further complicate comparisons among toxicological investigations. Consequently, reliable dose–response relationships remain difficult to establish for many EWNMs [142]. Development of internationally accepted testing protocols is therefore essential for generating reproducible data and supporting regulatory decision-making. EWNMs undergo continuous transformation following environmental release, including aggregation, dissolution, oxidation, reduction, sulfidation, and surface modification processes. These transformations can significantly alter environmental mobility, bioavailability, and toxicity. Traditional risk assessment approaches often assume relatively stable contaminant characteristics, whereas EWNMs may change substantially over time and across environmental compartments [128]. Such dynamic behavior introduces considerable uncertainty into exposure assessments and hazard evaluations. Accurate risk assessment therefore requires consideration of both primary nanoparticles and environmentally transformed products. Electronic waste environments contain complex mixtures of nanoparticles, heavy metals, persistent organic pollutants, flame retardants, plastic additives, and other contaminants. Interactions among these pollutants may result in additive, synergistic, or antagonistic effects that differ from those observed for individual contaminants. Current toxicological studies frequently evaluate single materials under controlled laboratory conditions, which may not accurately represent real-world exposure scenarios [143]. Improved understanding of mixture toxicity is therefore critical for realistic risk assessment and environmental management.

### 9.4. Occupational Safety Standards

Workers involved in electronic waste recycling represent one of the most highly exposed populations and therefore require effective occupational protection measures. Existing occupational exposure standards generally focus on airborne particulate matter, heavy metals, and chemical contaminants rather than nanoparticles specifically. Although several organizations have proposed exposure guidelines for selected engineered nanomaterials, nanoparticle-specific standards for EWNMs remain largely unavailable. Monitoring workplace nanoparticle concentrations is also challenging because conventional air quality measurements often fail to capture particle number concentrations and nanoscale fractions. Effective occupational protection requires implementation of engineering controls, local exhaust ventilation, enclosed processing systems, personal protective equipment, routine environmental monitoring, and worker health surveillance programs [144]. Special attention is needed in informal recycling sectors where occupational safeguards are frequently inadequate.

### 9.5. Need for Global Regulatory Harmonization

The transboundary nature of electronic waste generation, trade, recycling, and environmental contamination highlights the importance of international regulatory harmonization. Differences in national regulations, waste management infrastructure, monitoring capabilities, and enforcement practices create inconsistencies that may undermine environmental protection efforts. Developing countries often receive large quantities of electronic waste despite limited capacity for environmentally sound recycling and regulatory oversight. As a result, environmental releases of EWNMs may occur disproportionately in regions with weaker waste management systems [145]. Global harmonization should include standardized definitions, analytical methodologies, monitoring protocols, toxicological testing procedures, occupational exposure guidelines, and environmental quality criteria. International collaboration among governments, regulatory agencies, scientific organizations, and industry stakeholders will be essential for achieving these goals. Table 11 highlights that current international regulatory frameworks provide substantial guidance for electronic waste management and hazardous substance control; however, most regulations were developed before the emergence of concerns regarding electronic waste-derived nanomaterials. Existing policies primarily address bulk materials, hazardous chemicals, and waste transport, while the environmental behavior, exposure pathways, and toxicological characteristics of EWNMs remain insufficiently covered. Consequently, there is an increasing need for harmonized regulatory approaches that integrate nanomaterial-specific monitoring, risk assessment methodologies, occupational protection measures, and life-cycle management strategies for effective EWNM governance.

### 9.6. Future Regulatory Perspectives

Future regulatory strategies must evolve to address the unique challenges posed by EWNMs and other emerging nanoscale contaminants. Regulatory frameworks should move beyond conventional mass-based approaches and incorporate nanoparticle-specific metrics such as particle size distribution, surface area, particle number concentration, morphology, and surface reactivity. Advances in analytical technologies, environmental monitoring systems, computational toxicology, and artificial intelligence may facilitate development of more robust risk assessment methodologies [146]. Integration of life-cycle assessment approaches can further improve understanding of nanoparticle generation, environmental release, exposure pathways, and long-term impacts associated with electronic products.

**Figure 10:**
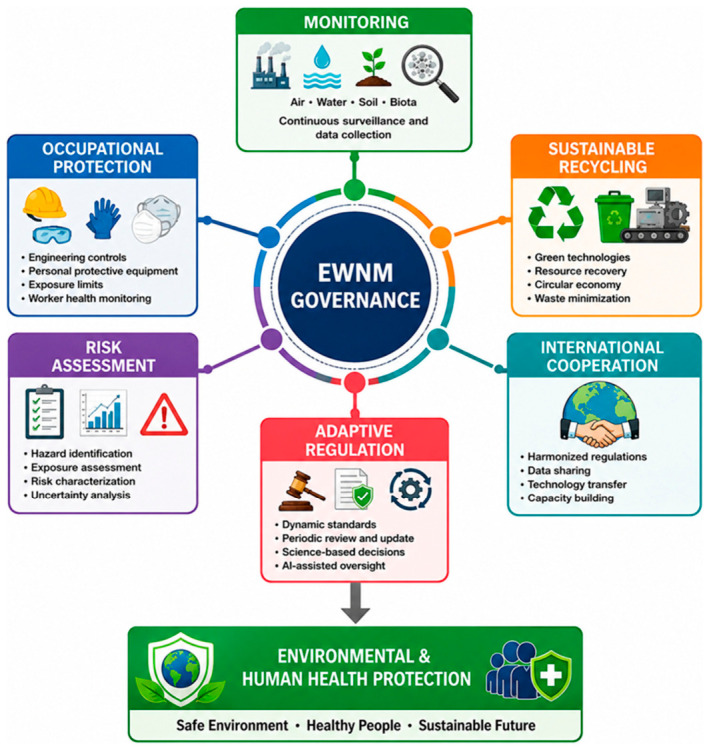
Conceptual framework illustrating the key components required for effective EWNM governance, including environmental monitoring, risk assessment, occupational protection, sustainable recycling technologies, international collaboration, and adaptive regulatory systems aimed at protecting environmental and public health.

Future regulations should also promote safer product design, sustainable recycling technologies, circular economy principles, and environmentally responsible resource recovery practices. A conceptual illustration is provided in Figure 10 for better understanding. Expanded producer responsibility programs, nanoparticle monitoring requirements, and mandatory occupational safety measures may contribute to reducing environmental and public health risks. Finally, adaptive regulatory frameworks capable of incorporating emerging scientific evidence will be critical for effective governance of EWNMs. Continued collaboration among researchers, policymakers, industry, and public health organizations will support the development of evidence-based regulations that balance technological advancement with environmental sustainability and human health protection. Future research should prioritize the development of cleaner recycling technologies that minimize nanoparticle generation while improving resource recovery efficiency. Greater efforts are also needed to establish standardized sampling and analytical protocols for consistent environmental monitoring and exposure assessment. Predictive models integrating particle transformation, transport, and long-term environmental fate should be developed to improve risk prediction across diverse ecosystems. In addition, life-cycle assessment approaches should be incorporated to evaluate the environmental impacts of electronic products from manufacturing to end-of-life management, thereby supporting more sustainable material design and circular economy strategies. Integrating these advances with multidisciplinary risk assessment frameworks will strengthen future environmental management and regulatory decision-making for EWNMs.

### 9.7. Techno-Economic Considerations for EWNMs Control Technologies

The successful implementation of technologies for controlling electronic waste-derived nanomaterials (EWNMs) depends not only on their technical performance but also on their economic feasibility and operational sustainability see in Table 12. While numerous physical, chemical, and biological treatment methods have demonstrated the ability to capture, remove, or transform nanoscale contaminants, their large-scale application is often constrained by capital investment, operating expenses, energy demand, maintenance requirements, and process scalability. Therefore, techno-economic evaluation has become an essential component in selecting appropriate EWNMs control strategies for recycling facilities, wastewater treatment systems, and contaminated environmental sites. Capital expenditure (CAPEX) represents the initial investment required for equipment procurement, installation, infrastructure development, and process integration. Conventional particulate control systems, including high-efficiency particulate air (HEPA) filters, electrostatic precipitators, and advanced membrane filtration units, generally require moderate to high initial investment because of specialized materials and engineering design. In contrast, process optimization measures, enclosure systems, and local exhaust ventilation can often be implemented with comparatively lower capital costs while providing substantial reductions in occupational nanoparticle exposure. The selection of a suitable technology therefore depends on facility size, waste processing capacity, and the expected concentration of EWNMs. Operating expenditure (OPEX) is equally important because it determines the long-term economic viability of pollution control systems. Major contributors include electricity consumption, replacement of filters or membranes, chemical reagents, labor, equipment maintenance, and waste disposal. Technologies requiring continuous chemical dosing or frequent replacement of consumable components generally incur higher operating costs than passive engineering controls. Consequently, achieving an optimal balance between treatment performance and operating cost is essential for sustainable implementation, particularly in regions where electronic waste recycling is dominated by small-scale or resource-limited facilities. Energy consumption is another important consideration because many advanced treatment technologies rely on pressure-driven filtration, thermal processing, plasma treatment, or electrochemical systems that require substantial energy input. Higher energy demand not only increases operating costs but may also offset environmental benefits by increasing indirect greenhouse gas emissions. Therefore, energy-efficient process design, waste heat recovery, and integration with renewable energy sources are increasingly recognized as practical approaches for improving the overall sustainability of EWNM control technologies. Treatment efficiency remains the primary technical criterion for evaluating pollution control performance. Depending on the selected technology and operating conditions, reported removal efficiencies for nanoscale particles frequently exceed 80–99% under optimized laboratory or pilot-scale conditions. However, actual field performance may vary because of fluctuations in particle size distribution, chemical composition, humidity, airflow, wastewater composition, and equipment operating conditions. Consequently, treatment efficiency should be evaluated together with process reliability and long-term operational stability rather than relying solely on laboratory-scale performance. The technology readiness level (TRL) also influences practical implementation. Established engineering controls such as HEPA filtration, electrostatic precipitation, dust collection systems, and conventional wastewater treatment processes possess relatively high TRLs and are already applied in industrial facilities. In contrast, several emerging approaches, including nanostructured adsorbents, photocatalytic degradation systems, advanced membrane materials, and hybrid treatment technologies, remain at laboratory or pilot scale and require additional validation before commercial deployment. Scalability and maintenance requirements further determine industrial applicability. Technologies that operate continuously with simple process control, minimal maintenance, and readily available replacement components are generally more suitable for large-scale recycling facilities. Conversely, systems requiring complex instrumentation, highly skilled operators, or frequent maintenance may face challenges during long-term operation despite demonstrating excellent removal performance under controlled conditions. Overall, no single technology simultaneously provides maximum removal efficiency, low capital and operating costs, minimal energy consumption, and high scalability. The most effective strategy is likely to involve integrated control systems that combine source reduction, engineering controls, efficient particle capture, and appropriate waste treatment according to site-specific conditions. Future research should emphasize comprehensive techno-economic assessments that incorporate life-cycle cost analysis, energy efficiency, environmental benefits, and operational reliability to support the development of sustainable and economically viable EWNMs management strategies.

## 10. Sustainable Management and Mitigation Strategies

The growing recognition of electronic waste-derived nanomaterials (EWNMs) as emerging environmental contaminants has highlighted the need for sustainable management strategies that minimize environmental release while maximizing resource recovery. Conventional electronic waste management practices often prioritize recovery of valuable metals without fully considering nanoparticle generation and associated environmental risks [39]. Consequently, future waste management systems must integrate environmental sustainability, occupational safety, circular economy principles, and advanced pollution prevention approaches. Effective mitigation of EWNM-related risks requires interventions throughout the entire electronic product life cycle, including product design, manufacturing, consumption, recycling, resource recovery, and final disposal. Sustainable strategies should focus not only on reducing waste generation but also on minimizing nanoparticle formation, environmental release, and human exposure during recycling and resource recovery activities.

### 10.1. Green Recycling Technologies

Green recycling technologies aim to recover valuable materials from electronic waste while minimizing environmental pollution, energy consumption, hazardous emissions, and nanoparticle release. Compared with conventional recycling methods, these approaches emphasize sustainability, resource efficiency, and environmental protection. Hydrometallurgical processing has emerged as an important alternative to traditional smelting and combustion-based recovery methods [147]. These techniques employ aqueous solutions to selectively dissolve and recover valuable metals from electronic waste. Compared with pyrometallurgical processes, hydrometallurgical methods generally operate at lower temperatures, resulting in reduced greenhouse gas emissions and lower generation of airborne nanoparticles. Common processes involve leaching, solvent extraction, ion exchange, precipitation, and electrowinning for recovery of metals such as copper, gold, silver, nickel, cobalt, and rare earth elements. Recent advances focus on developing environmentally benign leaching agents and closed-loop recycling systems that reduce secondary waste generation and improve recovery efficiency [148]. Bioleaching utilizes microorganisms to mobilize and recover metals from electronic waste through biological oxidation and dissolution processes. Various bacteria and fungi can generate organic acids, oxidizing agents, and metal-complexing compounds that facilitate metal extraction [149]. Compared with conventional acid leaching, bioleaching generally requires lower chemical inputs and produces fewer hazardous emissions. The technology is particularly attractive for recovery of copper, nickel, cobalt, zinc, and precious metals from complex electronic waste streams. Although bioleaching often requires longer processing times, ongoing research improves microbial efficiency, process optimization, and scalability for industrial applications. Electrochemical technologies have gained increasing attention for selective recovery of valuable metals from electronic waste recycling streams. Techniques such as electrodeposition, electrowinning, electrocoagulation, and electrochemical separation enable efficient recovery of metallic resources with relatively low environmental impact. Electrochemical approaches offer several advantages, including high selectivity, reduced chemical consumption, improved resource recovery efficiency, and compatibility with closed-loop recycling systems. These methods may also reduce the formation of secondary nanoparticle emissions associated with conventional thermal recovery processes. Thermal treatment remains an important component of electronic waste recycling; however, conventional combustion and smelting processes can generate significant quantities of airborne nanoparticles and hazardous emissions [150]. Modern low-emission thermal technologies seek to overcome these limitations through improved process control and emission reduction systems. Advanced pyrolysis, gasification, plasma processing, and controlled thermal decomposition technologies enable recovery of valuable materials while minimizing particulate emissions and environmental contamination. Integration of filtration systems, catalytic treatment units, and nanoparticle capture technologies further improves environmental performance.

### 10.2. Circular Economy Approaches

The circular economy provides a strategic framework for reducing electronic waste generation and minimizing environmental impacts associated with resource extraction and disposal. Rather than following the traditional linear model of production, consumption, and disposal, circular systems emphasize resource conservation, reuse, repair, refurbishment, remanufacturing, and recycling. Product design plays a critical role in determining the recyclability and environmental performance of electronic devices [151]. Design-for-recycling principles promote the use of modular architectures, simplified material compositions, standardized components, and easily separable materials. Improved product design facilitates efficient disassembly and material recovery while reducing the need for aggressive recycling processes that generate nanoparticles. The substitution of hazardous substances with environmentally safer alternatives may further reduce future EWNM formation. Extending product lifespan represents one of the most effective strategies for reducing electronic waste generation. Repairability, upgradeability, durability, and maintenance support can significantly delay product disposal and reduce demand for new electronic devices. Policies promoting repair rights, refurbishment programs, component replacement, and secondary markets may contribute to extending product lifecycles and reducing environmental burdens associated with frequent electronic replacement [152]. Urban mining refers to the recovery of valuable materials from existing products, waste streams, and discarded infrastructure. Electronic waste contains substantial quantities of precious metals, rare earth elements, and critical raw materials that can serve as secondary resource reservoirs. Efficient urban mining reduces dependence on primary mining activities, conserves natural resources, and decreases environmental impacts associated with raw material extraction. Sustainable urban mining systems can simultaneously support resource security and reduce uncontrolled EWNM releases.

### 10.3. Safe-by-Design Nanomaterial Strategies

The safe-by-design concept aims to reduce environmental and health risks by considering safety throughout material and product development stages. Although EWNMs are unintentionally generated, principles derived from safe-by-design approaches can be applied to electronic product manufacturing and recycling systems. Safer material selection, reduction in hazardous additives, substitution of toxic metals, and development of environmentally benign components may reduce the formation and toxicity of nanoparticles generated during product degradation and recycling [153]. Product designs that facilitate controlled disassembly and material recovery can further minimize nanoparticle release. Integration of toxicological assessment, life-cycle analysis, and sustainability evaluation during product development may contribute to safer electronic products and reduced environmental risks.

### 10.4. Nanoparticle Containment and Exposure Reduction

Reducing human and environmental exposure to EWNMs requires effective containment measures throughout recycling, processing, and waste management operations. Engineering controls represent the most effective means of reducing occupational nanoparticle exposure. Local exhaust ventilation systems, enclosed processing equipment, automated material handling systems, negative-pressure workspaces, and high-efficiency particulate air (HEPA) filtration systems can substantially reduce airborne nanoparticle concentrations. Process modifications that minimize dust generation and reduce manual handling of waste materials also contribute to exposure reduction. Personal protective equipment (PPE) provides an additional layer of protection for workers handling electronic waste [154]. Respirators, protective clothing, gloves, safety goggles, and specialized filtration masks can reduce inhalation and dermal exposure to nanoparticles. While PPE is important, it should be implemented as part of a broader exposure control strategy that prioritizes engineering controls and administrative measures. Environmental containment technologies help prevent the release of EWNMs into surrounding ecosystems. Air pollution control devices, wastewater treatment systems, dust suppression technologies, sealed storage facilities, and controlled disposal systems are critical components of environmentally responsible waste management. Advanced filtration systems, membrane technologies, adsorption processes, and nanoparticle capture methods may further reduce environmental emissions from recycling facilities.

### 10.5. Waste Minimization and Sustainable Manufacturing

Waste minimization strategies seek to reduce the quantity of electronic waste generated throughout product life cycles. Sustainable manufacturing practices emphasize efficient material use, reduced hazardous substance content, energy conservation, resource optimization, and environmentally responsible production methods. Material substitution, lightweight design, modular construction, and increased recyclability can reduce environmental burdens associated with electronic products [155]. Adoption of renewable energy sources and cleaner production technologies further contributes to sustainability objectives. Extended producer responsibility programs encourage manufacturers to consider end-of-life management during product development and promote accountability for waste generated by their products. Such approaches can incentivize innovation in sustainable product design and recycling technologies.

### 10.6. Policy Recommendations and International Cooperation

Effective management of EWNMs requires coordinated policy actions at local, national, and international levels. Existing regulations should be expanded to address nanoscale contaminants generated during electronic waste processing and environmental degradation. Key policy priorities include development of standardized nanoparticle monitoring protocols, implementation of occupational exposure guidelines, strengthening of environmental surveillance programs, promotion of green recycling technologies, and support for circular economy initiatives [156]. Regulatory frameworks should also encourage safer product design and adoption of cleaner manufacturing technologies. International cooperation is particularly important because electronic waste generation, trade, and environmental contamination frequently cross national boundaries. Collaboration among governments, industry, scientific institutions, international organizations, and public health agencies can facilitate harmonization of regulations, sharing of technical expertise, and development of global best practices. Capacity building in developing countries, technology transfer programs, financial support mechanisms, and international monitoring networks may further enhance sustainable electronic waste management and reduce environmental releases of EWNMs. Ultimately, integrating scientific evidence, technological innovation, and international cooperation will be essential for achieving environmentally sustainable and socially responsible management of electronic waste-derived nanomaterials. Table 13 outlines key policy priorities for the sustainable management of EWNMs across the entire electronic waste life cycle. Effective risk reduction requires the integration of regulatory measures, advanced recycling technologies, occupational protection strategies, environmental containment systems, and circular economy principles. Furthermore, international cooperation, standardized monitoring frameworks, and continued investment in research and innovation are essential for addressing current knowledge gaps and ensuring adaptive governance of emerging nanoscale contaminants derived from electronic waste.

## 11. Future Perspectives and Research Gaps

Electronic waste-derived nanomaterials (EWNMs) represent an emerging class of environmental contaminants whose environmental behavior, biological interactions, and long-term health implications remain incompletely understood. Although substantial progress has been made in understanding nanoparticle generation during electronic waste processing, major scientific uncertainties persist regarding environmental exposure, transformation mechanisms, toxicological responses, and regulatory management. Addressing these knowledge gaps will require interdisciplinary collaboration among environmental scientists, toxicologists, materials engineers, public health researchers, computational scientists, and policymakers. Future investigations should focus on developing realistic exposure models, advanced analytical tools, predictive risk assessment frameworks, and sustainable technological solutions capable of minimizing environmental and human health risks associated with EWNMs.

### 11.1. Need for Long-Term Toxicological Studies

Most currently available toxicological studies focus on short-term laboratory exposures conducted under controlled conditions. While these investigations provide valuable mechanistic insights, they may not accurately reflect chronic environmental exposure scenarios encountered by humans and ecological systems. Long-term studies are required to evaluate cumulative exposure effects, bioaccumulation, organ-specific toxicity, chronic inflammation, carcinogenicity, neurodegeneration, reproductive dysfunction, and transgenerational consequences associated with EWNMs. Particular attention should be given to vulnerable populations including children, pregnant women, elderly individuals, and recycling workers who may experience prolonged exposure [157]. Future research should also investigate low-dose chronic exposure conditions that better represent real-world environmental concentrations rather than relying solely on high-dose laboratory experiments.

### 11.2. Realistic Environmental Exposure Models

Current environmental risk assessments often rely on simplified laboratory exposure systems that may not accurately capture the complexity of natural environments. In reality, EWNMs undergo continuous transformation through aggregation, dissolution, oxidation, sulfidation, and interactions with natural organic matter. Future studies should develop environmentally relevant exposure models that account for variable physicochemical conditions, mixed pollutant interactions, seasonal fluctuations, and ecosystem-specific characteristics [158]. Such models should incorporate realistic concentrations and exposure durations to improve environmental risk predictions. Integrated field and laboratory investigations are needed to bridge the gap between controlled experimental studies and actual environmental conditions encountered in recycling regions and contaminated ecosystems.

### 11.3. Multi-Omics Approaches in Nanotoxicology

Recent advances in molecular biology provide powerful opportunities for understanding biological responses to EWNM exposure at multiple levels of biological organization [159]. Multi-omics technologies, including genomics, transcriptomics, proteomics, metabolomics, epigenomics, and microbiomics, can reveal complex molecular mechanisms underlying nanoparticle-induced toxicity. These approaches enable identification of biomarkers associated with oxidative stress, inflammation, immune dysfunction, metabolic disruption, neurotoxicity, and disease progression [160]. Multi-omics investigations can also improve understanding of dose–response relationships and susceptibility factors among different populations and species. Integration of multiple omics datasets through systems biology frameworks may provide a more comprehensive understanding of biological pathways affected by EWNM exposure and facilitate development of predictive toxicology models.

### 11.4. AI-Assisted Environmental Risk Prediction

**Figure 11:**
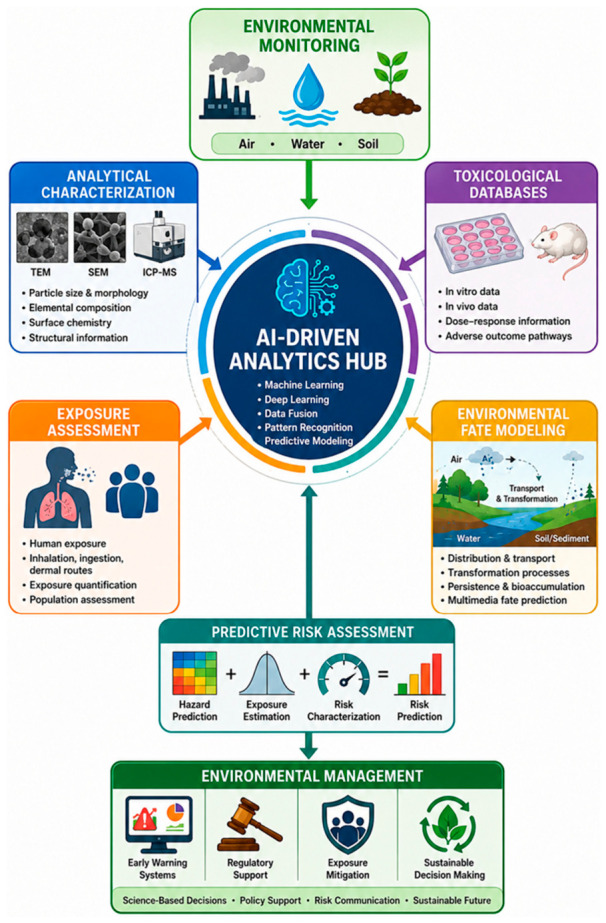
Conceptual framework illustrating the integration of environmental monitoring, analytical characterization, toxicological databases, exposure assessment, environmental fate modeling, and artificial intelligence tools for predictive risk assessment and evidence-based management of electronic waste-derived nanomaterials (EWNMs).

Artificial intelligence (AI), machine learning, and advanced computational modeling are expected to play increasingly important roles in EWNM research and risk assessment. Large datasets generated from environmental monitoring, toxicological experiments, spectroscopy, microscopy, and epidemiological studies can be analyzed using AI-based approaches to identify patterns and predict environmental risks. Figure 11 summarizes the key concepts discussed in this section. Machine learning algorithms can assist in predicting nanoparticle transport, transformation behavior, bioavailability, toxicity, and exposure pathways based on physicochemical characteristics [161]. AI-assisted predictive models may also support prioritization of high-risk contaminants and optimization of environmental monitoring programs. Future efforts should focus on developing transparent, validated, and interpretable AI frameworks supported by high-quality experimental data and standardized databases. 

### 11.5. Real-Time Monitoring Technologies

**Figure 12:**
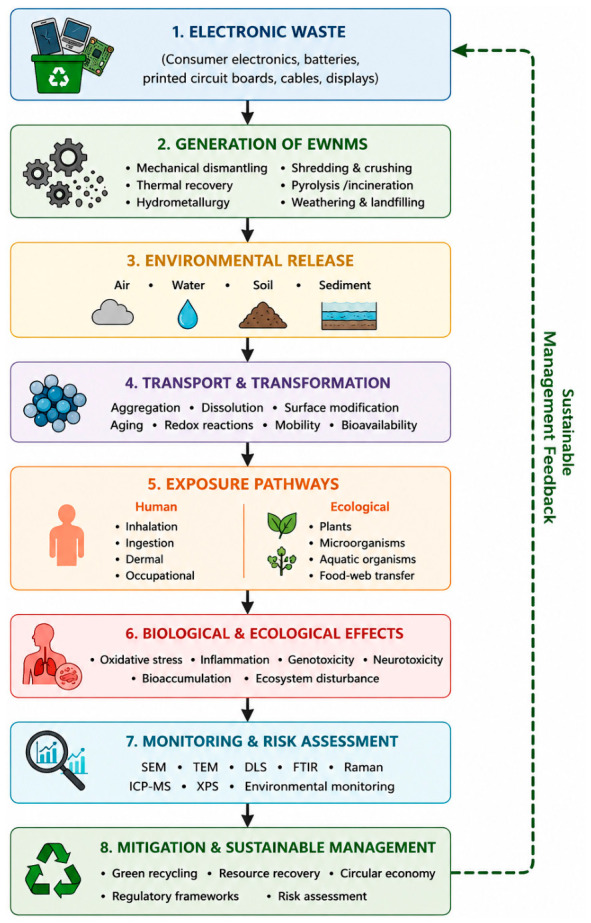
Integrated lifecycle framework of electronic waste-derived nanomaterials (EWNMs), illustrating their generation from electronic waste, environmental release, transport and transformation, human and ecological exposure pathways, biological impacts, monitoring and risk assessment, and sustainable management strategies.

One of the major limitations in current EWNM research is the lack of real-time monitoring capabilities for environmental and occupational exposure assessment. Most existing analytical methods require extensive sample preparation and laboratory-based analyses, limiting their suitability for continuous monitoring applications. Future technological developments should focus on portable sensors, smart monitoring platforms, biosensors, microfluidic devices, and internet-connected environmental surveillance systems capable of detecting nanoparticles in real time [162]. Such technologies could improve early detection of contamination events and support rapid risk management interventions. The integration of advanced sensors with wireless communication networks, cloud computing, and AI-assisted data processing may facilitate continuous environmental monitoring and exposure assessment across recycling facilities and contaminated regions. Figure 12 summarizes the complete lifecycle of electronic waste-derived nanomaterials (EWNMs), integrating their generation, environmental release, transport and transformation, exposure pathways, biological and ecological impacts, monitoring approaches, and sustainable management strategies discussed throughout this review.

### 11.6. Sustainable Electronic Material Development

Reducing future environmental risks associated with EWNMs requires innovation at the product design and manufacturing stages. Sustainable electronic materials that minimize hazardous substance content, improve recyclability, and reduce nanoparticle generation during disposal and recycling should become a major research priority. Emerging approaches include development of biodegradable electronic components, non-toxic alternatives to hazardous metals, environmentally benign flame retardants, and recyclable composite materials [163]. Design strategies that facilitate product disassembly, repair, reuse, and material recovery can further reduce environmental burdens associated with electronic waste. Life-cycle assessment should be incorporated into material development processes to evaluate environmental impacts throughout production, use, recycling, and disposal stages.

### 11.7. Translational Challenges from Laboratory to Real Environments

Despite significant advances in laboratory nanotoxicology, translating experimental findings into real-world environmental risk assessments remains a major challenge. Laboratory studies frequently employ simplified exposure systems, purified nanoparticles, and controlled environmental conditions that may not accurately represent environmental complexity. Environmental EWNMs exist as heterogeneous mixtures that interact with natural organic matter, microorganisms, co-contaminants, and variable physicochemical conditions [164]. Consequently, laboratory-derived toxicity data may not always predict environmental outcomes accurately. Future research should prioritize field validation studies, mesocosm experiments, long-term environmental monitoring programs, and integrated exposure assessment approaches capable of linking laboratory findings with real environmental scenarios.

### 11.8. Future Directions for Global E-Waste Governance

The transboundary nature of electronic waste generation and recycling necessitates coordinated global governance strategies capable of addressing both conventional pollutants and emerging nanoscale contaminants. Existing regulatory frameworks provide important foundations but remain insufficient for managing the unique challenges posed by EWNMs. Future governance efforts should focus on establishing internationally harmonized definitions, analytical standards, monitoring protocols, toxicity testing methodologies, exposure assessment frameworks, and environmental quality guidelines [165]. Strengthening international cooperation can facilitate information sharing, technology transfer, capacity building, and development of best management practices. Additional priorities include promoting circular economy principles, supporting green recycling technologies, enhancing occupational protection programs, and integrating nanoparticle considerations into existing electronic waste regulations. Collaborative efforts among governments, international organizations, industry stakeholders, and scientific communities will be essential for developing adaptive and evidence-based governance systems capable of addressing future challenges associated with EWNMs.

## 12. Conclusions

Electronic waste-derived nanomaterials (EWNMs) have emerged as a previously underrecognized class of environmental contaminants generated during the recycling, disposal, degradation, and weathering of electronic waste. Owing to their nanoscale dimensions, high surface reactivity, environmental mobility, and ability to interact with biological systems, these materials present unique environmental and public health challenges that differ significantly from those associated with conventional e-waste pollutants. This review highlights that EWNMs can be formed through multiple pathways, including mechanical fragmentation, thermal processing, chemical extraction, and environmental aging of electronic components. Once released, these nanomaterials can disperse through air, water, soil, and sediment systems, undergo complex physicochemical transformations, and become available to organisms across different trophic levels. Their capacity to induce oxidative stress, inflammation, genotoxicity, neurotoxicity, endocrine disruption, reproductive toxicity, and ecological disturbances underscores the need for comprehensive assessment of their potential risks. Despite increasing scientific attention, significant knowledge gaps remain regarding long-term environmental fate, realistic exposure scenarios, chronic toxicity, mixture effects, and population-level health outcomes. Current regulatory frameworks and risk assessment approaches are largely designed for bulk contaminants and are not fully equipped to address the complexities associated with nanoscale pollutants derived from electronic waste. Furthermore, limitations in analytical detection, environmental monitoring, and standardized testing methodologies continue to hinder accurate risk characterization. Future progress will require the integration of advanced analytical technologies, multi-omics approaches, artificial intelligence-assisted risk prediction, real-time monitoring systems, and environmentally relevant exposure models. Equally important are the development of sustainable electronic materials, green recycling technologies, circular economy practices, and internationally harmonized regulatory frameworks capable of minimizing nanoparticle generation and release. Overall, EWNMs represent an important emerging frontier at the intersection of environmental science, nanotechnology, waste management, toxicology, and public health. Addressing the challenges posed by these materials will require coordinated interdisciplinary research and global cooperation to ensure sustainable management of electronic waste while protecting environmental and human health in an increasingly technology-dependent world.

## Figures and Tables

**Table 1:** Global and Regional Electronic Waste Generation Statistics: Summary of global e-waste production trends, regional contributions, recycling rates, and projected future growth.

Indicator	Global/Regional Status	Key Findings	Environmental Significance	References
Global e-waste generation	Several million metric tons annually	E-waste is the fastest-growing solid waste stream worldwide	Increasing burden on waste management systems and environmental resources	[30,31,33]
Annual growth rate	Approximately few percent per year	Continuous increase driven by digitalization and technological turnover	Accelerates resource consumption and waste accumulation	[30,33]
Asia	Largest contributor to global e-waste generation	High population density and rapid technology adoption	Major source of recycling activities and environmental emissions	[30,32,33]
Europe	High per capita e-waste generation	Advanced collection and recycling infrastructure	Demonstrates benefits of regulated waste management systems	[32,33,35]
North America	Major producer of discarded electronic equipment	High consumption and replacement rates of electronic devices	Significant source of valuable secondary raw materials	[31,34]
Africa	Rapidly increasing e-waste generation	Growing imports of used electronics and limited recycling capacity	Elevated risk of informal recycling and environmental contamination	[32,35]
Latin America	Expanding electronic consumption	Developing collection and recycling networks	Increasing need for sustainable waste management policies	[32,35]
Global formal recycling rate	<25% of total e-waste generated	Large quantities remain undocumented or improperly managed	Contributes to environmental pollution and resource loss	[30,32,35]
Informal recycling sector	Predominant in many developing countries	Open burning, dismantling, and acid leaching widely practiced	Major source of hazardous emissions and nanoparticle generation	[35,39,44]
Projected e-waste generation by 2050	Expected to increase substantially	Driven by urbanization, digital transformation, renewable energy systems, and battery technologies	Increased potential for EWNM formation and environmental release	[30,33,35]

**Table 2:** Classification of electronic waste-derived nanomaterials (EWNMs).

EWNM Category	Primary Sources	Formation Pathways	Representative Physicochemical Properties	Environmental Relevance
Metal nanoparticles	Printed circuit boards, connectors, wiring, solder	Mechanical fragmentation, thermal volatilization, condensation	High electrical conductivity, high surface reactivity	Metal mobility, oxidative stress, bioaccumulation
Metal oxide nanoparticles	Batteries, semiconductors, electronic components	Oxidation during thermal recycling and environmental weathering	High catalytic activity, variable solubility	ROS generation, environmental persistence
Carbon-based nanomaterials	Plastics, cables, flame-retardant polymers, soot	Pyrolysis, combustion, incomplete burning	High surface area, strong adsorption capacity	Pollutant transport, contaminant adsorption
Semiconductor nanomaterials	Solar cells, LEDs, microchips, displays	Thermal degradation, mechanical fragmentation	Photoactive, semiconductor properties	Ecotoxicity, ion release
Polymer-derived nanoplastics	Cable insulation, electronic casings, polymer composites	Mechanical abrasion, UV degradation, environmental weathering	Hydrophobic surfaces, long-term persistence	Transport of organic pollutants and additives
Hybrid nanomaterials	Mixed electronic components	Combined mechanical, thermal and chemical processes	Mixed composition and heterogeneous surface chemistry	Complex environmental interactions and toxicity

**Table 3:** Summary of UV degradation, hydrolysis, oxidation, microbial transformation, and aging processes influencing EWNM formation and behavior.

Weathering Process	Primary Mechanism	Effect on EWNMs	Environmental Consequences	References
Ultraviolet (UV) Photodegradation	Exposure to solar UV radiation induces photochemical breakdown of polymers and electronic materials	Formation of secondary nanoparticles, surface oxidation, and structural fragmentation	Enhanced nanoparticle release and altered environmental mobility	[60,61]
Hydrolytic Weathering	Water-mediated cleavage of chemical bonds in polymers, coatings, and composite materials	Particle fragmentation, surface modification, and release of nanoscale constituents	Increased dispersion in aquatic and terrestrial environments	[61,64]
Corrosion-Induced Transformation	Electrochemical degradation of metallic electronic components	Release of metal-containing nanoparticles and ionic species	Enhanced metal mobility and environmental contamination	[62,65]
Oxidative Weathering	Reactions with atmospheric oxygen and reactive oxidants	Formation of metal oxides, altered surface chemistry, and changes in dissolution behavior	Modified toxicity, persistence, and environmental reactivity	[63,66]
Microbial Degradation	Microbial enzymatic activity acting on polymers and organic materials	Biotransformation of surface properties and nanoparticle release	Influences environmental fate and bioavailability	[62,67]
Surface Aging and Functionalization	Adsorption of natural organic matter, proteins, and environmental macromolecules	Formation of eco-coronas and modified surface charge	Alters transport, aggregation, and biological interactions	[63,66,67]
Aggregation During Aging	Particle-particle interactions promoted by environmental conditions	Formation of larger aggregates and agglomerates	Reduced mobility but increased sediment accumulation	[63,68]
Dissolution-Reprecipitation Processes	Release of metal ions followed by secondary particle formation	Generation of new nanoscale phases and mixed nanomaterials	Changes in bioavailability and ecotoxicological behavior	[62,65,66]
Combined Environmental Aging	Simultaneous action of UV radiation, moisture, oxidation, and biological activity	Progressive transformation of physicochemical properties	Dynamic changes in environmental persistence and toxicity	[60,61,62,63,64,66,68]

**Table 4:** Potential occurrence of electronic waste-derived nanomaterials (EWNMs) in different environmental matrices.

Environmental Matrix	Dominant EWNM Composition	Possible Environmental Significance
Air (recycling workshops)	Cu, Pb, Ni, Cr, Sn, Fe nanoparticles; metal oxides	High occupational inhalation exposure and atmospheric transport
Air (open burning sites)	Metal oxides, soot, carbonaceous nanoparticles, polymer-derived particles	Elevated emission of ultrafine particles with high respiratory deposition potential
Surface soil	CuO, ZnO, Fe_2_O_3_, Pb-containing nanoparticles, nanoplastics	Long-term accumulation, reduced soil quality, plant uptake
Sediment	Metal oxides, semiconductor particles, carbonaceous nanomaterials	Acts as a long-term sink for EWNMs and associated contaminants
Surface water	Metal nanoparticles, metal oxides, nanoplastics	Aquatic transport, aggregation, and potential uptake by aquatic organisms
Landfill leachate	Dissolved metal colloids, metal oxide nanoparticles, polymer-derived nanoplastics	Migration into groundwater and surrounding aquatic environments

**Table 5:** Summary of soil deposition mechanisms, interactions with soil organic matter, and plant uptake pathways governing the terrestrial distribution, mobility, and bioavailability of EWNMs.

Process Category	Mechanism	Influence on EWNM Behavior	Environmental Implications	References
Atmospheric Deposition	Dry and wet deposition of airborne EWNMs onto soil surfaces	Introduces nanoparticles into terrestrial ecosystems	Primary pathway for soil contamination near recycling and disposal sites	[64,66]
Particulate Settling	Sedimentation of suspended nanoscale particles from air	Promotes accumulation in surface soils	Increases local contaminant concentrations	[68,73]
Surface Adsorption	Attachment of EWNMs to soil minerals and clay particles	Reduces mobility and enhances retention	Influences environmental persistence	[63,67]
Interaction with Soil Organic Matter	Association with humic and fulvic substances	Alters surface charge and colloidal stability	Modifies transport and bioavailability	[63,67,73]
Organic Coating Formation	Adsorption of natural organic compounds onto particle surfaces	Changes aggregation and dispersion behavior	Affects environmental fate and biological interactions	[67,73,74]
Soil Pore Transport	Movement through pore water and soil matrices	Enables redistribution within soil profiles	Influences exposure of soil biota and plants	[63,64,75,76]
Root Surface Adsorption	Binding of EWNMs to root epidermal tissues	Facilitates initial plant interaction	Determines uptake efficiency	[67,75]
Root Uptake	Absorption through root systems and vascular tissues	Enables internal accumulation within plants	Entry route into terrestrial food webs	[64,73,75]
Translocation to Aerial Tissues	Movement from roots to stems, leaves, and edible tissues	Expands distribution within plants	Potential pathway for dietary exposure	[64,73,75]
Bioaccumulation in Plant Biomass	Progressive accumulation during growth	Increases trophic transfer potential	Raises concerns regarding food-chain contamination	[64,73,75,77]
Aggregation and Agglomeration	Particle-particle interactions promoted by soil constituents	Increases effective particle size	Reduces mobility but enhances local accumulation	[63,68,73,78]

**Table 6:** Summary of the major physicochemical properties governing nanoparticle dissolution, transformation behavior, environmental stability, and persistence, including particle size, surface characteristics, crystallinity, aggregation behavior, and interactions with surrounding environmental conditions.

Physicochemical Factor	Influence on Dissolution Behavior	Effect on Environmental Persistence	Environmental Significance	References
Particle Morphology	Shape-dependent surface exposure influences dissolution kinetics	Determines transport and transformation behavior	Alters environmental interactions and biological uptake	[78,83]
Particle Size	Smaller particles exhibit greater surface area and faster dissolution rates	Reduced persistence but increased release of ionic species	Enhances bioavailability and potential toxicity	[83,87]
Specific Surface Area	High surface area promotes surface-mediated reactions and ion release	Accelerates environmental transformation processes	Influences reactivity and environmental fate	[83,87]
Surface Charge (Zeta Potential)	Regulates interactions with dissolved ions and colloids	Affects colloidal stability and mobility	Controls aggregation and persistence in environmental media	[78,83]
Chemical Composition	Metal and metal oxide composition governs solubility characteristics	Influences persistence and transformation pathways	Determines environmental risk profile	[83,87]
Surface Functionalization	Surface coatings modify dissolution and adsorption processes	Enhances or reduces environmental stability	Affects mobility and bioavailability	[78,83,87]
Crystallinity and Phase Structure	Highly crystalline materials generally dissolve more slowly	Increases environmental persistence	Influences long-term stability and transformation rates	[83,85]
Aggregation and Agglomeration	Aggregated particles exhibit reduced exposed surface area	Slows dissolution and enhances sediment accumulation	Alters transport and environmental distribution	[78,84,86]
Natural Organic Matter Interactions	Organic coatings can inhibit or promote dissolution	Modifies persistence and environmental mobility	Controls colloidal behavior in natural waters and soils	[84,86,87]
Environmental pH	Acidic conditions often accelerate metal ion release	Reduces nanoparticle stability	Major driver of transformation and bioavailability	[82,83,87]
Redox Conditions	Oxidative and reductive environments alter particle chemistry	Influences dissolution and secondary particle formation	Governs environmental transformation pathways	[83,87]
Ionic Strength and Electrolytes	Dissolved salts affect aggregation and surface reactivity	Modifies environmental persistence and transport	Important determinant of fate in aquatic systems	[78,84,86]

**Table 7:** Environmental occurrence, persistence, bioaccumulation, and toxicological characteristics of representative electronic waste-derived nanomaterials (EWNMs).

EWNM Type	Primary Environmental Compartment	Persistence/Environmental Behavior	Bioaccumulation Potential	Major Toxicological Effects	Representative Test Organism/Cell Line	Ref.
Cu/CuO nanoparticles	Air, soil, water	Moderate persistence; oxidation and dissolution	Moderate to high	Oxidative stress, membrane damage, DNA damage	Human lung cells (A549), zebrafish	[87,89]
ZnO nanoparticles	Soil, wastewater	Partial dissolution with Zn^2+^ release	Moderate	ROS generation, cytotoxicity, inflammation	Human epithelial cells, algae	[87,89,90]
Carbonaceous nanoparticles	Air, sediment	Highly persistent	Moderate	Oxidative stress, inflammatory responses	Macrophages, fish	[87,89,91]
Pb-containing nanoparticles	Air, soil	High persistence	High	Neurotoxicity, developmental toxicity	Mammalian cells, rodents	[87,92]
Polymer-derived nanoplastics	Water, soil, sediment	Very high persistence	Moderate to high	Cellular uptake, inflammation, pollutant carrier effects	*Daphnia magna*, zebrafish, intestinal cells	[87,93,94]
Semiconductor nanoparticles (Si, GaAs, In-containing)	Soil, sediment	Moderate to high persistence	Moderate	Cytotoxicity, oxidative stress	Human cell lines, microbial models	[87,90,92]

**Table 8:** Summary of DNA damage mechanisms, chromosomal alterations, and epigenetic effects reported for electronic waste-derived nanomaterials.

Genotoxic Endpoint	Underlying Mechanism	Biological Consequences	Representative EWNMs	References
DNA Strand Breaks	ROS-mediated oxidative damage and direct interaction with DNA molecules	Single- and double-strand DNA breaks, genomic instability	Metal-containing nanoparticles, metal oxides	[91,94]
Oxidative DNA Damage	Excessive generation of reactive oxygen species (ROS)	Formation of oxidized DNA bases and impaired DNA integrity	Cu-, Pb-, Ni-, and Co-containing EWNMs	[91,102]
DNA Adduct Formation	Interaction of released metal ions with nucleic acids	Structural modification of DNA and altered replication fidelity	Metal-rich EWNMs	[92,102]
Chromosomal Aberrations	DNA damage and mitotic disruption	Chromosome fragmentation, deletions, and rearrangements	Mixed-metal nanoparticles	[98,99]
Micronucleus Formation	Incomplete chromosome segregation during cell division	Indicator of chromosomal instability and mutagenicity	Metallic and metal oxide EWNMs	[98,99]
Mitotic Spindle Disruption	Interaction with microtubule assembly and cell-cycle machinery	Aneuploidy and abnormal chromosome distribution	Nanoscale metal particles	[90,99]
Cell Cycle Arrest	Activation of DNA damage response pathways	Inhibition of cell proliferation and repair mechanisms	Diverse EWNM classes	[94,95]
Mitochondrial DNA Damage	ROS generation and mitochondrial dysfunction	Impaired cellular energy metabolism and apoptosis	Metal oxide nanoparticles	[91,95]
Epigenetic Modifications	Alteration of DNA methylation and histone regulation	Changes in gene expression without DNA sequence alteration	Persistent EWNM exposure	[92,102]
Altered microRNA Expression	Nanoparticle-induced regulation of non-coding RNAs	Dysregulation of cellular signaling and stress responses	Mixed-composition EWNMs	[92,100]
Impaired DNA Repair Mechanisms	Suppression of DNA repair enzymes and pathways	Increased accumulation of genetic lesions	Reactive metal-containing EWNMs	[94,102]
Apoptosis Following Genotoxic Stress	Persistent DNA damage triggering programmed cell death	Tissue injury and loss of cellular function	Various EWNMs	[91,95]

**Table 9:** Key knowledge gaps and future research priorities associated with exposure assessment, epidemiological investigations, biomonitoring, occupational health protection, and risk management of electronic waste-derived nanomaterials.

Public Health Challenge	Current Limitation	Potential Consequences	Research Priority	References
Occupational Exposure Assessment	Limited quantitative data on workplace EWNM concentrations	Underestimation of worker exposure risks	Development of standardized exposure monitoring protocols	[110,124]
Inhalation Exposure Characterization	Insufficient information on nanoparticle deposition and clearance	Uncertainty in respiratory risk assessment	Long-term inhalation toxicology studies	[112,121]
Dermal Exposure Evaluation	Limited understanding of skin penetration mechanisms	Incomplete assessment of occupational exposure pathways	Mechanistic studies on dermal absorption and barrier disruption	[115,127]
Dietary Exposure Assessment	Scarce data on food-chain transfer and dietary intake	Potential underestimation of chronic exposure	Investigation of trophic transfer and food contamination pathways	[106,118]
Biomonitoring Strategies	Lack of validated biomarkers of EWNM exposure and effect	Difficulty in identifying exposed populations	Development of sensitive biomarkers and biomonitoring frameworks	[109,126]
Epidemiological Evidence	Limited long-term population-based studies	Weak linkage between exposure and disease outcomes	Large-scale cohort and longitudinal studies	[110,124]
Vulnerable Population Assessment	Insufficient data for children, pregnant women, and elderly individuals	Increased uncertainty regarding susceptibility	Targeted exposure and health impact studies	[104,120]
Respiratory Health Evaluation	Inadequate assessment of chronic pulmonary effects	Delayed recognition of respiratory diseases	Long-term respiratory surveillance programs	[114,121]
Cardiovascular Risk Characterization	Limited mechanistic and epidemiological evidence	Uncertain cardiovascular disease burden	Investigation of nanoparticle-induced vascular dysfunction	[119,122]
Neurological Health Assessment	Insufficient evidence regarding chronic neurotoxicity	Incomplete understanding of neurological risks	Studies on neuroinflammation and neurodegenerative outcomes	[109,126]
Cancer Risk Evaluation	Lack of long-term carcinogenicity data	Uncertain cancer risk estimation	Lifetime exposure and carcinogenicity studies	[123,126]
Exposure Standards and Guidelines	Absence of EWNM-specific occupational exposure limits	Inconsistent risk management practices	Establishment of evidence-based exposure thresholds	[126,127]
Risk Communication	Limited awareness among workers and local communities	Reduced effectiveness of preventive measures	Development of community-focused risk communication programs	[110,126]
Global Risk Governance	Regulatory fragmentation among countries	Inconsistent public health protection	International harmonization of monitoring and regulatory frameworks	[109,126]

**Table 10:** Comparative evaluation of analytical techniques used for the characterization of electronic waste-derived nanomaterials (EWNMs).

Technique	Primary Information Obtained	Typical Detection Range/Resolution	Advantages	Limitations	Environmental Applicability
SEM	Surface morphology, particle size	~1–10 nm resolution	Rapid imaging, elemental analysis with EDS	Limited internal structure	Airborne particles, soil, dust
TEM	Internal structure, morphology, crystallinity	<1 nm resolution	Ultra-high resolution	Complex sample preparation	Individual nanoparticles
AFM	Surface topography, roughness	Sub-nanometer vertical resolution	Three-dimensional surface analysis	Small scan area	Surface-bound nanoparticles
XRD	Crystal structure, phase identification	Crystalline materials	Reliable phase analysis	Low sensitivity for amorphous materials	Metal and metal oxide nanoparticles
DLS	Hydrodynamic particle size	~1 nm–10 µm	Rapid particle sizing in suspension	Sensitive to aggregation	Water and colloidal samples
FTIR	Functional groups, surface chemistry	Molecular vibrations	Identifies organic coatings	Limited elemental information	Nanoplastics and surface-modified particles
Raman spectroscopy	Molecular composition, carbon structure	Micrometer scale	Minimal sample preparation	Fluorescence interference	Carbonaceous materials, polymers
ICP-MS	Trace elemental composition	ng L^−1^ to pg L^−1^	Extremely high sensitivity	Destructive analysis	Water, soil extracts, biological samples
XPS	Surface elemental composition, oxidation state	Surface depth ~5–10 nm	Surface chemical analysis	Vacuum requirement	Surface-modified nanoparticles
Synchrotron-based techniques	Elemental speciation, oxidation state, nanoscale mapping	Very high spatial resolution	Advanced chemical speciation	Limited facility availability	Complex environmental matrices

**Table 11:** Summary of major international agreements, directives, regulations, and policy frameworks governing electronic waste management, hazardous substances, resource recovery, occupational safety, and emerging nanomaterial-related environmental concerns.

Regulatory Framework	Geographic Scope	Primary Focus	Key Provisions Relevant to E-Waste and EWNMs	Major Limitations for EWNMs	References
Basel Convention	International	Transboundary movement of hazardous waste	Controls international transport and environmentally sound management of hazardous electronic waste	Limited provisions specifically addressing nanoscale contaminants	[139,140]
Basel Ban Amendment	International	Restriction of hazardous waste exports	Prohibits export of hazardous waste from developed to developing countries for disposal	Does not explicitly regulate EWNM generation and environmental release	[139,140]
European Union WEEE Directive	European Union	Collection, recycling, and recovery of e-waste	Establishes producer responsibility, collection targets, and recycling requirements for electrical and electronic equipment	Primarily addresses bulk waste streams rather than nanoscale materials	[140,141]
European Union RoHS Directive	European Union	Restriction of hazardous substances	Restricts the use of Pb, Hg, Cd, Cr(VI), PBBs, and PBDEs in electronic equipment	Limited consideration of nanoparticle-specific hazards	[140,141]
REACH Regulation	European Union	Chemical registration and risk assessment	Requires registration, evaluation, authorization, and restriction of chemical substances, including increasing attention to nanomaterials	Nanomaterial-specific requirements continue to evolve	[126,128]
Stockholm Convention	International	Persistent organic pollutants (POPs)	Restricts production and use of hazardous organic pollutants, including flame retardants present in electronic waste	Does not directly regulate EWNMs generated during recycling	[128,141]
Minamata Convention on Mercury	International	Mercury pollution control	Reduces mercury use, emissions, and releases from products and waste streams	Limited consideration of mercury-containing nanoparticles formed during waste processing	[128,141]
OECD Nanomaterial Safety Program	International	Nanomaterial testing and risk assessment	Develops standardized testing methods and safety assessment frameworks for manufactured nanomaterials	Not specifically developed for e-waste-derived nanomaterials	[126,141]
ISO Nanotechnology Standards	International	Standardization and characterization	Provides standardized terminology, characterization methods, measurement protocols, and safety guidance for nanomaterials	Limited application to heterogeneous EWNM mixtures in environmental systems	[130,132]
United Nations Sustainable Development Goals (SDGs)	Global	Sustainable production and environmental protection	Promotes circular economy, responsible consumption, sustainable waste management, and resource efficiency	Non-binding framework with no specific EWNM regulations	[140,145]
National E-Waste Regulations (Various Countries)	National	E-waste collection and recycling management	Establish extended producer responsibility (EPR), recycling infrastructure, and disposal requirements	Regulatory implementation and enforcement vary considerably among countries	[139,140]
Occupational Safety Regulations (OSHA, EU-OSHA, etc.)	National/Regional	Worker health protection	Establish workplace exposure controls, personal protective equipment (PPE), hazard communication, and occupational monitoring	Few nanoparticle-specific occupational exposure limits currently exist	[142,144]
Emerging Nanomaterial Governance Frameworks	International/National	Nanotechnology risk management	Promote life-cycle assessment, nanosafety evaluation, environmental monitoring, and risk-based governance	Lack harmonized protocols for EWNM-specific environmental risk assessment	[126,128]

**Table 12:** Comparative techno-economic assessment of representative EWNMs control technologies.

Technology	Typical Removal Efficiency (%)	CAPEX	OPEX	Energy Demand	TRL	Scalability	Maintenance Requirements	Advantages	Limitations
High-Efficiency Particulate Air (HEPA) filtration	99.0–99.97	High	Moderate	Moderate	High (TRL 9)	High	Periodic filter replacement	High removal efficiency for ultrafine particles; well-established technology	Filter clogging, pressure drop, recurring replacement costs
Electrostatic Precipitators (ESP)	90–99	High	Low-Moderate	Moderate	High (TRL 9)	High	Routine electrode cleaning	Continuous operation, low pressure drop, suitable for industrial emissions	Reduced efficiency for very small particles under variable operating conditions
Membrane Filtration (UF/NF/RO)	90–99	High	High	High	High (TRL 8–9)	Moderate	Membrane cleaning and replacement	Effective removal of nanoparticles and dissolved contaminants	Membrane fouling, high energy demand, concentrate disposal
Adsorption (Activated carbon/Biochar/Nanomaterials)	70–98	Low-Moderate	Moderate	Low	Medium-High (TRL 7–9)	High	Periodic adsorbent regeneration or replacement	Simple operation, relatively low energy consumption	Adsorbent saturation, secondary waste generation
Coagulation-Flocculation	70–95	Low	Low	Low	High (TRL 9)	High	Chemical dosing and sludge handling	Cost-effective and widely implemented in wastewater treatment	Reduced efficiency for stable nanoparticles; sludge management required
Advanced Oxidation Processes (AOPs)	75–98	High	High	High	Medium (TRL 6–8)	Moderate	Oxidant handling and reactor maintenance	Degrades persistent organic contaminants while transforming nanoparticles	High operational cost and energy consumption
Phytoremediation/Bioremediation	40–80	Low	Low	Very Low	Medium (TRL 5–7)	Moderate	Periodic biomass management	Environmentally friendly and sustainable	Slow treatment rate; limited effectiveness for heavily contaminated sites
Integrated Hybrid Systems	>95	High	Moderate-High	Moderate-High	Medium-High (TRL 7–8)	High	Depends on system complexity	Combines multiple treatment mechanisms for enhanced performance	Greater system complexity and higher initial investment

Abbreviations: CAPEX, capital expenditure; OPEX, operating expenditure; TRL, technology readiness level; UF, ultrafiltration; NF, nanofiltration; RO, reverse osmosis; ESP, electrostatic precipitator; AOPs, advanced oxidation processes. Removal efficiencies are representative ranges reported in the literature and may vary depending on particle characteristics, operating conditions, and treatment configuration.

**Table 13:** Policy Priorities for Sustainable EWNM Governance. Overview of recommended measures for environmental protection, occupational safety, circular economy implementation, and responsible management of EWNMs.

Policy Area	Priority Action	Expected Outcome	Sustainability Significance	References
Regulatory Framework Development	Establish EWNM-specific regulations and risk assessment guidelines	Improved identification and control of nanoscale contaminants	Strengthens environmental and public health protection	[39,151]
Standardized Monitoring Programs	Develop harmonized protocols for EWNM sampling, characterization, and environmental surveillance	Reliable exposure and risk evaluation	Supports evidence-based decision-making	[39,148]
Green Recycling Technologies	Promote low-emission recycling, bioleaching, and environmentally benign recovery processes	Reduced nanoparticle generation and pollutant release	Enhances resource recovery and environmental sustainability	[147,149]
Circular Economy Implementation	Encourage product redesign, reuse, repair, and material recovery	Reduced e-waste generation and resource consumption	Improves long-term material sustainability	[147,150]
Safe-by-Design Manufacturing	Integrate environmental safety considerations into electronic product development	Lower environmental release of hazardous nanomaterials	Supports sustainable innovation	[39,151]
Occupational Health Protection	Strengthen workplace exposure controls, engineering measures, and PPE utilization	Reduced worker exposure to EWNMs	Improves occupational safety in recycling facilities	[148,152]
Environmental Containment Systems	Implement advanced filtration, emission control, and wastewater treatment technologies	Minimized environmental dispersion of EWNMs	Protects terrestrial and aquatic ecosystems	[149,153]
Public Awareness and Education	Promote stakeholder engagement and responsible disposal practices	Increased participation in formal recycling programs	Reduces informal recycling activities	[150,152]
Research and Innovation Support	Increase funding for EWNM toxicology, environmental monitoring, and mitigation technologies	Improved scientific understanding and risk management tools	Facilitates sustainable policy development	[39,151]
International Collaboration	Promote global harmonization of regulations, technology transfer, and data sharing	Consistent management of transboundary e-waste challenges	Strengthens global environmental governance	[147,150]
Extended Producer Responsibility (EPR)	Expand producer accountability for end-of-life electronic products	Improved collection and recycling efficiency	Encourages sustainable product life-cycle management	[147,150]
Adaptive Policy Frameworks	Regularly update regulations based on emerging scientific evidence and technological advances	Responsive and effective EWNM governance	Supports long-term environmental resilience	[151,153]

## Data Availability

No new data were created or analyzed in this study. Data sharing is not applicable to this article.
